# Understanding youth and young adult cannabis use in Canada post-legalization: a scoping review on a public health issue

**DOI:** 10.1186/s13011-024-00615-9

**Published:** 2024-06-17

**Authors:** Toula Kourgiantakis, Ragave Vicknarajah, Judith Logan, Travonne Edwards, Eunjung Lee, Shelley Craig, Ashima Kaura, Charmaine C. Williams, Savannah Marshall

**Affiliations:** 1 École de travail social et de criminologie, Université Laval, 1030, avenue des Sciences-Humaines, Pavillon Charles-De-Koninck (local 6489), Québec, QC G1V 0A6 Canada; 2 Factor-Inwentash Faculty of Social Work, University of Toronto, Toronto, ON Canada; 3 John P. Robarts Library, University of Toronto, Toronto, ON Canada; 4 School of Child and Youth Care, Toronto Metropolitan University, Toronto, ON Canada

**Keywords:** Cannabis, Legalization, Regulation, Youth, Young adults, Canada, Public health, Scoping review

## Abstract

**Background:**

Canada legalized recreational cannabis in 2018, and one of the primary objectives of the Cannabis Act was to protect youth by reducing their access to cannabis and providing public education. Canada has the highest prevalence of cannabis use worldwide, particularly among youth and young adults under the age of 25. Cannabis use is linked with many adverse effects for youth and young adults including psychosis, anxiety, depression, respiratory distress, cannabinoid hyperemesis syndrome, and impaired cognitive performance. Despite the high prevalence of cannabis use and the evolution of policies in Canada and globally, significant knowledge and research gaps remain regarding youth and young adult cannabis use. The aim of this scoping review is to map the extent, nature, and range of evidence available on youth and young adult cannabis use in Canada since its legalization, in order to strengthen policies, services, treatments, training, and public education strategies.

**Methods:**

Using a scoping review framework developed by Arksey and O’Malley, along with the PRISMA-ScR guidelines, we conducted a rigorous search in five academic databases: MEDLINE, Embase, APA PsycINFO, CINAHL and Web of Science Core Collection. We included empirical studies that collected data in Canada after the legalization of recreational cannabis (October 2018) and focused on youth or young adults < 30. Two reviewers independently screened articles in two stages and extracted relevant information from articles meeting the inclusion criteria.

**Results:**

Of the 47 articles meeting our inclusion criteria, 92% used quantitative methods, 6% were qualitative, and 2% used a mixed-methods approach. Over two-thirds (68%) used secondary data. These studies were categorized into six focus areas: (1) prevalence, patterns, and trends, (2) cannabis-related injuries and emergency department (ED) visits, (3) rates and patterns during the pandemic, (4) perceptions of cannabis use, (5) prevention tools, and (6) cannabis-related offenses. Key findings from the studies reviewed include an increase in cannabis use among 18-24-year-olds post-legalization, with mixed results for youth under 18. ED visits for intentional and unintentional cannabis-related injuries have increased in young children and teens. Perception studies show a mix of concern and normalization of cannabis use. Though limited, prevention studies are promising in raising awareness. A decline in cannabis-related offenses was noted by one study. The review highlights several research gaps, including the need for more qualitative data, disaggregation of demographic data, intervention research, and comprehensive studies on the physical and mental health impacts of cannabis use among youth and young adults.

**Conclusion:**

Maintaining a public health approach is critical, with a focus on reducing the high prevalence of cannabis use among youth and young adults. This involves implementing prevention strategies to minimize harms, enhancing public education, minimizing commercialization, reducing youth access to cannabis, promoting guidelines for lower-risk cannabis use and harm reduction strategies, and increasing training for healthcare providers.

**Supplementary Information:**

The online version contains supplementary material available at 10.1186/s13011-024-00615-9.

## Introduction

Canada ranks among the top countries globally for cannabis use prevalence [1]. According to the Canadian Cannabis Survey (CCS), the highest usage rates are in young adults aged 20–24, with 51% reporting past-year use. This is followed by 37% of those aged 16–19, compared to 21% of adults over the age of 25 [2]. Daily cannabis use, which is linked to more significant adverse effects, is reported by 16% of 16-19-year-olds and 23% of those aged 20 to 24 [2]. The CCS also indicates that 66% of bisexual and 58.3% of gay or lesbian young adults reported past-month use, compared to 45% of heterosexual young adults. In terms of racial identity, youth and young adults who identify as white have the highest rates of cannabis use [2].

Canada legalized recreational cannabis in 2018, with the nationwide policy aiming to regulate the sale and distribution of cannabis. One of the primary objectives of the Cannabis Act was to protect public health and safety, particularly focusing on protecting young people by reducing incentives to use cannabis and restricting access [3]. However, since legalization, 45.5% of those aged 16–19 and 49.8% of 20-24-year-olds have reported increased cannabis consumption [2]. Furthermore, youth and young adults have reported easier access to cannabis since its legalization [4, 5]. Notably, 41% of students in grades 7–12 in Ontario – Canada’s most populous province, reported easy access to cannabis in the Ontario Student Drug and Health Survey (OSDHUS) [6].

Cannabis use among youth and young adults remains a serious public health concern, associated with various adverse mental health outcomes. These include poor wellbeing and psychosocial functioning [7, 8], psychosis [9–15], anxiety [8, 16], and depression [7, 8, 17, 18]. Cannabis is also linked to physical health issues, including impaired cognitive performance, respiratory distress, lung injury, myocardial ischemia, seizures, weight loss, and cannabinoid hyperemesis syndrome [19]. There is also a notable trend of polysubstance use among young people [20], often involving alcohol and vaping [21]. Early initiation and frequent cannabis use are correlated with other issues [11, 22], including elevated risks of suicidality [23] and cannabis dependence [24].

A significant proportion of youth and young adults use cannabis products with tetrahydrocannabinol (THC) levels over 20%, a stark increase from the 3% levels seen in the 1980s [2]. THC is the main psychoactive ingredient in cannabis, and higher potencies are more harmful, especially for young people. In a position paper written pre-legalization and reaffirmed in 2023, the Canadian Paediatric Society (CPS) recommended limiting THC concentration in legally purchased cannabis for young adults aged 18–25. The CPS explains that there are higher risks associated with cannabis use during this important developmental period, as the brains of young adults have not fully developed [25].

The data show a correlation between young people’s self-reports of their mental health and their cannabis use. For example, among 16-19-year-olds, 44.9% of those who rated their mental health as poor reported using cannabis in the past 12 months, compared to 24.4% of those who rated their mental health as excellent. Similarly, in the 20-24-year-old group, 34.5% who considered their mental health excellent used cannabis, but this ratenearly doubled to 67.3% among those who perceived their mental health as poor [2]. A systematic review and meta-analysis by Leung et al. [26] found that 22% of individuals who use cannabis have cannabis use disorder, 13% abuse cannabis, and another 13% have cannabis dependence, with higher risks in young people and those who are using weekly or daily.

Despite the widespread use of cannabis and evolving policies in Canada and around the world, significant knowledge and research gaps persist regarding cannabis use among youth and young adults in Canada since legalization [27]. A number of systematic and scoping reviews have examined cannabis use and legalization, though they are not specifically focused on Canada [28–32] or not focused on youth and young adult populations since legalization [26, 33, 34]. Among those reviews focusing on youth or young adults, most are not specific to Canada and concentrate on specific areas such as driving [35], cognitive functioning [36], co-occurring cannabis use with other substances or behaviours [37], and psychosocial interventions for cannabis reduction in young adults with psychosis [38]. Rubin-Kahana and colleagues [39] explored the impact of cannabis legalization on youth in Canada, but this was a narrative review that did not adhere to systematic or scoping review guidelines and did not have a list of studies selected for the review. To our knowledge, there are no systematic or scoping reviews that have been conducted since cannabis legalization in Canada that focus specifically on youth and young adults. This type of review is relevant and necessary especially at this early stage of legalization while the government assesses the public health effects, particularly the impact on youth and young adults [33, 39, 40].

A public health approach to the regulation of cannabis has been recognized as an important strategy that focuses on minimizing harms and promoting health [33, 41]. It is an evidence-based approach, relying on the best available scientific evidence to inform policy and practice decisions [40, 41]. To address some of the gaps identified in the literature, this scoping review aims to map the extent, nature, and range of evidence available on youth and young adult cannabis use in Canada post-legalization to strengthen policies, services, treatments, training, and public education strategies. Our review focused on the following research questions: (1) What types of literature are available that describe youth and young adult cannabis use in Canada since its legalization? (2) What evidence does the literature provide regarding youth and young adult cannabis use following its legalization in Canada? (3) What are the identified limitations, gaps, and recommendations in the literature concerning youth and young adult cannabis use since its legalization?

## Methods

This scoping review followed the framework established by Arksey and O’Malley [42], which comprises five key stages: (1) defining research questions, (2) identifying relevant articles, (3) selecting and incorporating pertinent material, (4) charting data, and (5) synthesizing and reporting the findings. In the final phase, the authors conducted a narrative synthesis of the results, presenting them through written summaries and descriptive charts. A narrative synthesis identifies patterns, similarities, and differences across studies [42], allowing the authors to organize the literature thematically in different categories. Scoping reviews primarily aim to synthesize and map existing literature related to a specific research topic, without focusing on appraising the methodological rigor of the selected material [43]. The rationale, objectives, and methods of this scoping review were detailed in a peer-reviewed scoping review protocol to enhance methodological transparency [44]. We adhered to the standards outlined in the PRISMA Extension for Scoping Reviews (PRISMA-ScR) checklist [45], which is available as a supplemental document (Appendix A).

### Identifying relevant articles

A member of the research team who is a social sciences librarian (JL) designed a comprehensive search of the published literature. Text words and controlled vocabulary relating to youth, cannabis, and Canada were adapted from reputable published search strings [46–49]. A pilot search strategy, developed for Medline (Ovid), underwent peer review by an independent librarian using the Peer Review of Electronic Search Strategies (PRESS) framework [50]. This pilot strategy was then translated into Embase (Ovid), APA PsycINFO (Ovid), CINAHL (EBSCO), and Web of Science Core Collection, which includes Science Citation Index Expanded, Social Sciences Citation Index, Arts & Humanities Citation Index, Emerging Sources Citation Index, Conference Proceedings Citation Index, and Book Citation Index. Search results were downloaded on December 8, 2021, and uploaded to Covidence for deduplication and screening. The search was rerun on July 14, 2022, and March 8, 2023, to capture articles published or added to the databases since the initial download, yielding 149 and 229 studies for screening, respectively. A date limit of the year 2000 + was applied to all searches. After the first update on July 14, 2022, we revised the inclusion criteria to exclude results published before the legalization of cannabis in Canada in 2018. However, we decided not to change the publication date limit in the next update on March 8, 2023, opting instead to manually screen out studies published between 2000 and October 2018 that had not already been excluded. The full search strategy in five databases is available as a supplemental document (Appendix B).

### Article selection and inclusion

Articles were eligible for inclusion in this study if they (1) were written in English or French, (2) were empirical studies published in peer-reviewed journals, (3) focused on recreational cannabis use in youth and young adults aged 29 and younger, 4) were conducted in Canada, 5) collected data post-legalization of recreational cannabis on October 17, 2018. The most recent search we conducted was in March 2023, and we included articles up to that point. We excluded gray literature, dissertations, editorials, commentaries, books, book chapters, book reviews, interviews, newspapers, conference abstracts, and reviews. Articles with participants aged 30 and over were included if there was also a subsample of youth or young adults under the age of 30. Throughout this review, the term “youth” refers to anyone under the age of 18 and “young adult” refers to anyone under the age of 30. The Cannabis Act refers specifically to “young persons” as those under the age of 18 [3]. However, Health Canada [41], the Canadian Paediatric Society [25], the Mental Health Commission of Canada (MHCC) [51], and many researchers have discussed concerns about cannabis use in young adults aged 18–24, a group with the highest prevalence of cannabis use and who are in a period of continued brain development. For this scoping review, we included up to age 29 aligning with the MHCC which states that this stage of development can extend up to age 30 [51]. Our aim was to ensure we do not miss research studies due to a narrow age range for young adults and a preliminary literature scan showed that some studies had young adult samples up to age 25, while others went up to age 29.

Articles focused on substances other than cannabis or on polysubstance use were excluded, unless cannabis was specifically mentioned as one of the substances of interest in the abstract. Articles on the medical use of cannabis, as well as those that did not collect data in Canada, were excluded. However, studies collecting data from multiple countries were included if Canadian data were separately analyzed and reported. Trained members of our research team (RV, TE, AK, SM, TK) applied these inclusion and exclusion criteria and screened the articles in two stages. The first stage involved screening by title and abstract, while the second stage entailed a full-text review of articles that met inclusion criteria in the first stage.

### Charting data

The subsequent stage of the review involved charting and extracting data from articles that met inclusion criteria. The charting categories were aligned with our research questions and included: (1) author(s), (2) title, (3) year, (4) language, (5) province/territory, (6) journal, (7) aim of study, (8) research method, (9) research design, (10) sample size and characteristics, (11) key findings related to cannabis, (12) gaps or limitations, and (13) recommendations. This phase followed an iterative approach. Four members of the research team (TK, RV, SM, AK) conducted a comprehensive review of all selected articles, utilizing a charting template to synthesize and analyze the key findings from these articles [52].

## Results

**Fig. 1:**
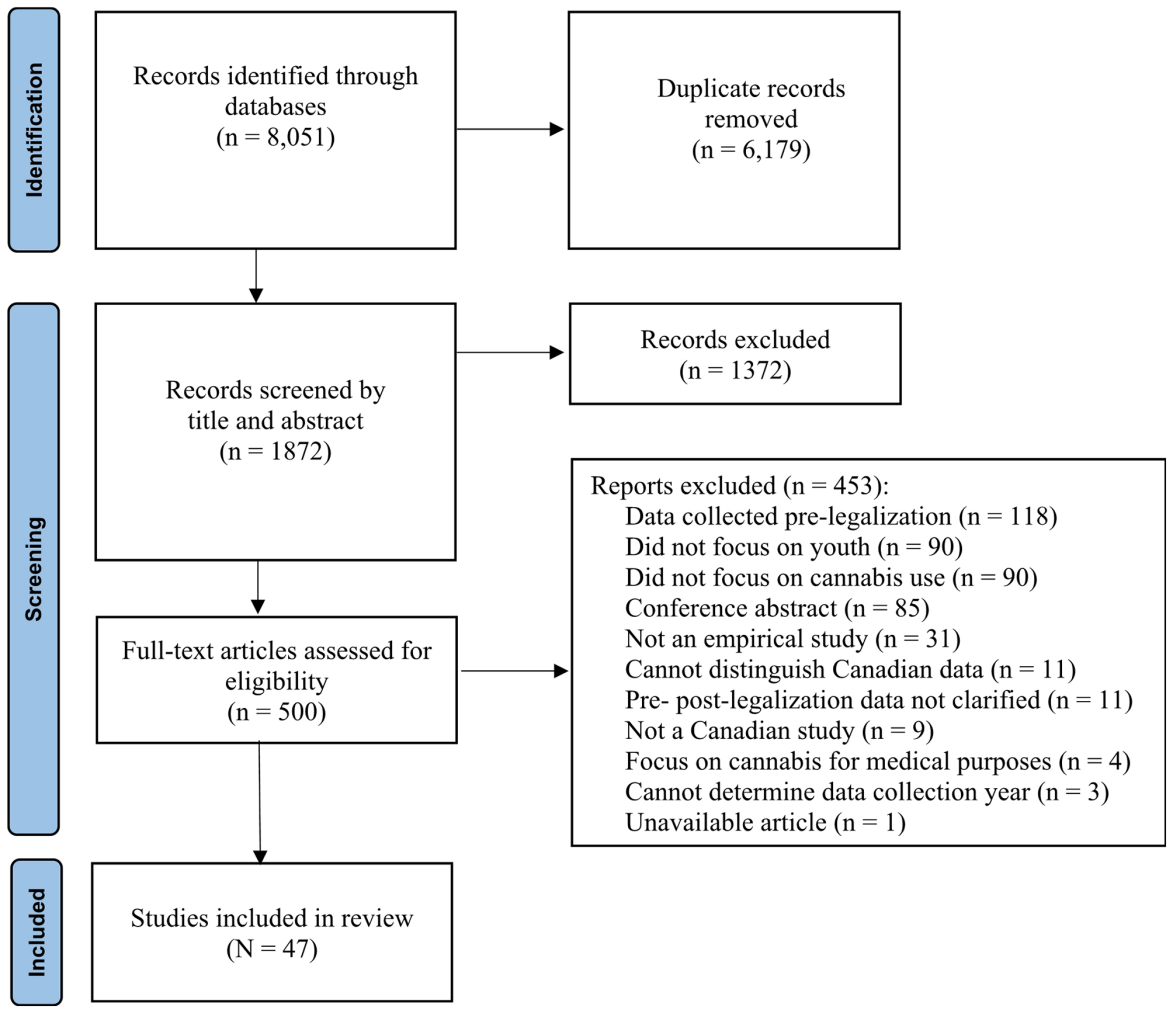
PRISMA flowchart of the search and screening process

An initial database search in December 2021 identified 3,326 article titles. A second search conducted in July 2022 yielded 768 articles, and a third and final search in March 2023 resulted in 3,957 article titles. In total, 8,051 articles were identified from the selected databases. After removing duplicates (*n* = 6,179), 1,872 articles remained from all three searches. During the title and abstract screening phase, 1,372 articles were excluded for not meeting the eligibility criteria. The full-text screening phase involved reviewing 500 articles, leading to the exclusion of 453 articles that did not meet the eligibility criteria, resulting in a final sample of 47 articles that met all eligibility criteria. Figure 1 presents the PRISMA flowchart of the screening and search process.

### Study characteristics

All studies collected data in Canada, with some specifying particular provinces while others did not. Eleven studies either covered all or most provinces or did not specify their locations within Canada [17, 53–62]. Eight studies focused on the most populated provinces: Québec, Ontario, Alberta, and British Columbia [63–70]. Two studies collected data across two provinces: Leatherdale et al. [71] in Ontario and Québec, and Callaghan, Sanches et al. [72] in Ontario and Alberta. Additionally, one study focused on undergraduates in Atlantic Canada, though the sample included students from other provinces [73]. Two studies included data from both Canada and the United States [74, 75] and one included data from Canada, the United States, and England [76]. The remaining studies gathered data from a single province, with eleven studies in Ontario [77–86, 95], four in Québec [4, 15, 20, 87], two in Manitoba [88, 89], two in British Columbia [90, 91], and one each in Saskatchewan [92], Alberta [93], and Newfoundland and Labrador [94].

Most studies employed quantitative research methods (*n* = 43, 92%), with only three studies (6%) utilizing qualitative methods [73, 91, 94], and one (2%) using a mixed method approach [80]. Over two thirds of the studies (*n* = 32, 68.09%) used secondary data from various sources, with the most frequently cited being the COMPASS study (*n* = 9, 19.15%) which is a longitudinal study following Canadian high school students.

Across the 47 studies, 21 (44.68%) had participants aged 18 or younger [55, 60, 62–72, 77, 78, 86, 90, 92–95], seven studies (14.89%) had samples of young adults aged 18–29 [17, 20, 54, 57, 61, 73, 88], and eleven studies (23.40%) included both youth and young adult participants aged 0–29 [4, 15, 76, 79–82, 85, 87, 89, 91]. The remaining eight studies (17.02%) did not focus specifically on youth or young adults but included subgroups or presented results for younger age groups [53, 56, 58, 59, 74, 75, 83, 84].

Sample sizes varied: eight studies had 0–100 participants [17, 53, 62, 73, 77, 80, 91, 94], two had 101–300 [74, 79], five had 301–500 [54, 58, 61, 90, 92], four had 501–1000 [64, 67, 88, 89] and the remaining 28 studies had more than 1000 participants. Demographic information was inconsistently provided by the studies. Just over two-thirds of studies (*n* = 32) provided information on the gender of participants, but only 11 offered three or more gender categories, including transgender and nonbinary [17, 54, 57, 65, 66, 78, 79, 82, 91, 92, 94]. Fewer than one-third of the studies (*n* = 15) reported the race or ethnicity of participants, with four categorizing participants as “white and other” without providing specific ethnoracial information [59, 61, 75, 76]. Additionally, none of the studies included information on the sexual orientation of participants. Table 1 presents characteristics of selected studies.

**Table 1:** Characteristics of Selected Studies (*N* = 47)

Characteristic	Studies *n* (%)
Data collection location	
Canada (province unspecified or includes most provinces/territories)	11 (23.4%)
Two or more provinces	11 (23.4%)
Ontario	11 (23.4%)
Québec	4 (8.51%)
Canada & other countries (i.e., United States & England)	3 (6.38%)
Manitoba	2 (4.26%)
British Columbia	2 (4.26%)
Saskatchewan	1 (2.13%)
Alberta	1 (2.13%)
Newfoundland & Labrador	1 (2.13%)
Method	
Quantitative	43 (91.49%)
Qualitative	3 (6.38%)
Mixed Methods	1 (2.13%)
Use of secondary data	32 (68.09%)
Sample sizes	
≤ 100	10 (21.28%)
101–300	2 (4.26%)
301–500	2 (4.26%)
501–1000	6 (12.77%)
≥ 1001	27 (57.45%)
Studies that did not focus specifically on youth and/or young adults	8 (17.02%)
Categories by Area of Study Focus	
Prevalence rates, patterns/trends, and characteristics	17 (36.17%)
Cannabis use rates post-legalization (*n* = 7), patterns & trends (*n* = 10)	
Cannabis-related injuries and ED visits	12 (25.53%)
Cannabis use rates & patterns during the COVID-19 pandemic	9 (19.15%)
Perceptions related to cannabis use	6 (12.77%)
Prevention tools (psychosis & cannabis-impaired driving)	2 (4.26%)
Cannabis-related offences	1 (2.13%)

*Note* Many articles were not limited to a single subject area, but they were categorized based on the primary focus of the study

### Categories by areas of study focus

In our review, we grouped the 47 studies into six categories based on their focus areas. The first category, with the highest number of studies (*n* = 17), examined the prevalence, patterns, trends, and characteristics of cannabis use among youth and young adults. This category is further divided into two subgroups: the first, with seven studies, focused on changes in cannabis use rates post-legalization [4, 15, 56, 60, 69, 76, 88] and the second subgroup, comprising ten studies, on patterns, trends, and characteristics of cannabis use in youth and young adults since legalization [63, 65, 67, 68, 70, 75, 79, 85, 92, 95].

The second category encompassed twelve studies examining cannabis-related injuries and emergency department (ED) visits attributable to cannabis [53, 62, 64, 72, 74, 77, 81, 83, 84, 86, 87, 93]. While most studies in this category examined the rates of ED visits since legalization, two examined trends related to vaping [53, 62], and one study examined traffic injury presentations related to cannabis in EDs [72]. It is important to note that although two studies examined hospital visits during the COVID-19 pandemic [81, 86], we grouped them with the ED visits, rather than the COVID-19 category because the primary focus was ED visits and cannabis-related injuries.

The third category, consisting of nine studies, specifically focused on cannabis use rates and patterns during the COVID-19 pandemic [17, 20, 58, 59, 61, 66, 71, 78, 89]. The fourth category, includes nine articles exploring perceptions related to cannabis use: four focus on youth and young adult perceptions post-legalization [57, 73, 90, 94] one examines perceptions of substance use services [91] and another explores perceptions of cannabis policies and penalties related to immigrants and prejudices [54].

The fifth category consists of two articles focused on prevention tools. The first is aimed at preventing cannabis-impaired driving [82], and the second focuses on increasing awareness among Black youth and young adults about cannabis and psychosis through a psychoeducational video game [80]. Finally, the sixth category consists of a single article that focuses on cannabis-related offences since legalization [55]. For a full list of selected articles and a synthesis of results, see Table 2.

**Table 2:** Full set of selected articles and synthesis of results (*N* = 47)

Authors & Year	Study Location	Sample size and population	Research Method	Category & aim of study	Findings
Andrews et al. (2022)	Canada, Alberta & United States (US)	*N* = 157 patients (*n* = 50 Alberta, *n* = 50 Canada, *n* = 57 US) Subgroup of larger sample aged 16–24 Gender: female (53%), male (47%) Race/ethnicity: Latino (5.3%), African (1.8%), East Asian (1.8%), white (77%) etc.	Quantitative Secondary data	Category 2 – Cannabis related injuries & emergency department (ED) visits Examined characteristics of patients with Cannabinoid Hyperemesis Syndrome (CHS) and treatment prevalence of CHS before and during the COVID-19 pandemic in Alberta, as well as pre and post legalization.	Treatment prevalence for CHS was highest among 16–24-year-olds, with cases in EDs doubling from 2017 to 2021. Most CHS patients also met criteria for cannabis use disorder. In Alberta, CHS treatment rates for this age group doubled between 2018 and 2021.
Auger et al. (2021)	Québec	*N* = 12,858 hospitalizations for youth 0 to 20 + years presenting to the ER Gender: N/A Race/ethnicity: N/A	Quantitative Secondary data	Category 2 – Cannabis related injuries & ED visits Investigated trends in cannabis-related hospitalizations among youth pre and post legalization of recreational cannabis in Canada.	Post-legalization, cannabis-related hospitalizations increased in boys under 15, but not in girls or older boys. Cannabis was reported in 70% of substance-related hospitalizations in boys 10–14 post-legalization compared with 39% pre-legalization.
Baker et al. (2022)	Canada (provinces were not specified)	*N* = 20 vaping-associated lung illness (VALI) patients aged 15 to 50+ Gender: N/A Race/ethnicity: N/A	Quantitative Secondary data	Category 2 – Cannabis related injuries & ED visits Explored the demographic and clinical patient characteristics of vaping-associated lung illness (VALI) cases in Canada from 2019 to 2020.	25% of VALI cases involved youths aged 16 to 19. VALI patients reported using cannabis-related vaping or dabbing products. All patients under 20 who reported vaping using both nicotine- and cannabis-containing products, were youth under 20. 80% of VALI patients required hospitalized.
Bartel, & Stewart (2020)	Canada (provinces were not specified)	*N* = 70 young adults who were part of a longitudinal study aged 19–25 years Gender: female (45%), male (24%), other (1%) Race/ethnicity: N/A	Quantitative	Category 3 – Cannabis use rates & patterns during the pandemic Investigated whether self-isolation and depression motives would predict cannabis use levels during the pandemic.	Young adults who self isolated used 20% more cannabis. Those who consistently used cannabis to cope with depression during the pandemic consumed 31% more. Over 75% of the sample either smoked (59%) or vaped cannabis (17%).
Bishop et al. (2022)	Newfound- land & Labrador	*N* = 38 youth aged 13 to 18 Gender: girl (64%), boy (34%), transgender (2%) Race/ethnicity: N/A	Qualitative	Category 4 – Perceptions related to cannabis use Explored youths’ perceptions of their cannabis health literacy and future educational needs.	Youth are seeking more evidence-informed cannabis education that incorporates harm -reduction principles. They advocate for the early integration of cannabis education in school curricula, and increased programming in schools and on social media.
Buliga & MacInnis (2022)	Canada (provinces were not specified)	*N* = 380 undergraduate students with mean age of 21 Gender: women (73.9%), men (25.5%), nonbinary (0.5%) Race/ethnicity: East Asian (12.63%), South Asian, (12.37%), Filipino, (6.84%), Black, (4.21%), 54.21% white etc. Citizenship: 25% Naturalized Canadians, 75% Canadian-born citizens	Quantitative	Category 4 – Perceptions related to cannabis use Examined the relationship between intergroup contact and support for cannabis-related policies and penalties specific to immigrants.	The study found that greater interaction with immigrants among undergraduate participants was linked to lower support for harsh cannabis-related penalties for immigrants. High-quality contact with immigrants was associated with stronger opposition to such penalties. Furthermore, individuals with right-wing ideologies tended to support more severe penalties for outgroup members (immigrants) compared to ingroup members (Canadians in general).
Butler et al. (2022)	Ontario	*N* = 28, 219 elementary and high school students (14–18 years of age) and *N* = 60 secondary schools in wave 4 of study (2018/19) Gender: N/A Race/ethnicity: N/A	Quantitative Secondary data	Category 1 – Prevalence rates, patterns & trends Examined health topic priorities of schools and how schools rank alcohol and other drug use (AOUD) as a priority during the period of cannabis legalization.	Mental health was the top priority in most Ontario secondary schools, followed by bullying and substance use. Priorities remain unchanged before and after cannabis legalization, and the schools’ focus on substance use was not associated with student cannabis and alcohol use behaviours.
Callaghan, Sanches et al. (2021)	Alberta & Ontario	Ontario: *N* = 4565 youth drivers’ ED visits (aged 16–18) Gender: female visits (49.2%) Alberta: *N* = 3265 youth drivers’ ED visits (aged 14–17) Gender: female visits (54.7%) Race/ethnicity: N/A	Quantitative Secondary data	Category 2 – Cannabis related injuries & ED visits Evaluated the associations between cannabis legalization and traffic-injury emergency department presentations.	There were no significant changes in drivers’ traffic injury ED visits following legalization.
Callaghan, Vander Heiden et al. (2021)	Canada (provinces were not specified)	*N* = 41 179 youth with cannabis-related offenses between 12–17 years of age Gender: female (22%), male (78%) Race/ethnicity: N/A	Quantitative Secondary data	Category 6 – Cannabis-related offenses Examined whether the Cannabis Act was associated with changes in police-reported cannabis-related offences among youth in Canada.	There were significant short term reductions of 55–65% in police-reported cannabis offences among youth aged 12–17.
Coret & Rowan-Legg (2022)	Ontario	*N* = 37 charts of children < 18 at a pediatric hospital with mean age of 5.9 (range 0.9 to 17) Gender: male (*n* = 22), female (*n* = 15) Race/ethnicity: N/A	Quantitative Secondary data	Category 2 – Cannabis related injuries & ED visits Examined unintentional cannabis exposures in children pre- and post-legalization.	Post-legalization, there were 32 cases of child/youth exposure compared to 5 pre-legalization. Accidental exposures, primarily due to edible products, accounted for 76% of incidents. The most common abnormal vital sign was tachycardia. The most frequent signs and symptoms were altered level of consciousness (76%), lethargy (59%), vomiting (30%), and seizures (22%). Patient dispositions were observation in ED (68%), inpatient admission (30%), and ICU admission (2%). Cannabinoid toxicology tests were performed in 49% of cases.
Dumas et al. (2020)	Ontario	*N* = 1054 youth 14–18 years of age Gender: female (76.4%), male 21.9%, nonbinary/gender fluid 1.2%, N/A (0.6%) Race/ethnicity: Asian (15.3%), Black North American/African (3.9%), Latino (3.1%), white/European (65.7%), other (11%), N/A (0.9%)	Quantitative	Category 3 – Cannabis use rates & patterns during the pandemic Examined changes in adolescent substance use including cannabis during the COVID-19 pandemic.	The frequency of cannabis use increased significantly from pre-COVID to post-COVID.
Fischer et al. (2021)	Canada (provinces were not specified)	Sample sizes unavailable. Data from National Cannabis Survey (NCS), Canadian Cannabis Survey (CCS), Canadian Student Tobacco, Alcohol, and Drugs Survey (CSTADS), and International Cannabis Policy Study Gender: N/A Race/ethnicity: N/A	Quantitative Secondary data	Category 1 – Prevalence rates, patterns & trends Examined population level data and key indicators to assess impacts from pre- to post-legalization of cannabis in Canada.	Cannabis use increased across all age groups, with the highest increase among 20–24-year-olds. The most frequent use (daily or near daily) occurred in the 18–24-year-old group, with 16.3% using daily or near daily. Smoking remained the most common mode of consumption. In 2020, the rate of obtaining cannabis from illegal sources was highest among 18–24-year-olds at 56.6%. Additionally, 40% of respondents under the legal age reported that it was very easy to obtain cannabis.
Gueye et al. (2021)	Manitoba	*N* = 951 undergraduate students 18–24 years of age Gender: female (70.6%), male (29.4%) Race/ethnicity: Black (16.5%), Métis (10.4%), Arab (5.1%), Asian (3.6%), other (2.4%), white (62%)	Quantitative	Category 1 – Prevalence rates, patterns & trends Compared self-reported cannabis use pre- and post-legalization among university students.	Cannabis use among students who used it regularly (3 times or more in the last month) increased from 46.7% in 2012, to 47.7% in 2018, and 53.8% in 2019. Students with less frequent use, either in the past month or year, showed an increase immediately after legalization, followed by a decrease. Most participants used recreational cannabis only (84.6%), and 15.4% used it for both recreational and medical purposes. No participants used cannabis solely for medical purposes.
Hammami & Katapally (2022)	Saskatch-ewan	*N* = 401 youth aged 13 to 18 Gender: female (56.1%), male (38.2%), transgender or prefer not to disclose (5.7%) Race/ethnicity: Indigenous (5%), Canadian (40%), other ethnicities (54.5%)	Quantitative	Category 1 – Prevalence rates, patterns & trends Investigated the role of subjective health, internalizing and externalizing behaviours including cannabis use in the association between victimization and suicide ideation among youth.	Cannabis prevalence rates among study participants were lower than the national average (25.3% versus 44.8%), yet cannabis use was identified as a behavioural risk factor associated with suicidal ideation.
Hammond et al. (2022)	Canada & US (provinces were not specified)	*N* = 27,024 in 2018 *N* = 45,426 in 2019, *N* = 45,180 in 2020 respondents aged 16 to 65 Gender: female (50%), male (50%) Race/ethnicity: white 78%, other/mixed (22%)	Quantitative Secondary data	Category 1 – Prevalence rates, patterns & trends Examined use of different cannabis products and trends in consumption patterns in Canada and the US between 2018–2020.	Dried flower is the dominant cannabis product in Canada. Pre-rolled joints, vape pens, and edibles are the most commonly used forms among young people. Males were more likely to report more frequent and daily use. Young adults aged 21–35 years reported the highest consumption across most product forms.
Hammond et al. (2021)	Canada, England, United States (provinces were not specified)	Samples of 16–19-year-olds in Canada (*n* = 11,779), United States (*n* = 11,869), and England (*n* = 11,117) Gender (Canadian participants): male (51%), female (49%) Race/ethnicity (Canadian participants): white only 2017 (59%), 2018 (47%), 2019 (55%)	Quantitative Secondary data	Category 1 – Prevalence rates, patterns & trends Examined changes in cannabis use and modes of consumption among youth in Canada, England, and the United States.	Prevalence rates for the past 30 days and past 12 months were higher in Canada and the US than in England. In Canadian youth, prevalence increased between 2017 and 2019 for daily use, past month, and past 12 months from 22.5% in 2017, 23.4% in 2018, and 27% in 2019.
Harris-Lane et al. (2023)	Canada (all provinces and territories except Yukon)	*N* = 1424 young adults 18 to 25 years of age and most were students (87.5%) Gender: woman (67.7%), man (25.9%), nonbinary (2.4%), Transgender (0.4%), Multiple gender identities (2.5%), gender fluid (0.4%), N/A (0.8%) Race/ethnicity: Asian/South Asian (11.1%), Black/African-Canadian (2.9%), Middle Eastern (2.3%), East Indian (2.1%), Latino (1.0%), multiple identities (5.6%), white (72.6%) etc.	Quantitative	Category 4 – Perceptions related to cannabis use Explored emerging adult perceptions of cannabis consumption and if perceptions changed based on age and sex.	Young adults perceived almost daily cannabis consumption as a “small” to “moderate” problem, and often underestimated their usage. Frequent cannabis use was not perceived differently across genders. Young adults appeared to recognize the harms of cannabis for younger adolescents but not for themselves. They perceived differences in harm between 21-year-olds and 28-year-olds.
Hawke, & Henderson (2021)	Ontario	*N* = 101 recruited prior to legalization and *N* = 168 post-legalization Participants were < 19 or 19+ Gender: man/boy (43.8%), woman/girl (54.2%), transgender/gender diverse (2.1%) Race/ethnicity: Black (8.1%), Asian (8.1%), Latin American (3.0%), Indigenous (3.0%), Caucasian (57.6%) etc.	Quantitative	Category 1 – Prevalence rates, patterns & trends Examined cannabis use profiles of youth seeking substance use services before and after the legalization of cannabis in Ontario, Canada.	Youth reported high cannabis use both pre-legalization (75.3%) and post-legalization (79.3%). Before legalization, 10.9% believed they were purchasing from a government source, compared to 35.7% post-legalization. The majority of youth reported using cannabis alone both before and after legalization. Polysubstance use was highly pervasive in the sample. There were few significant differences between pre- and post-legalization periods.
Imtiaz et al. (2022)	Canada (all provinces and territories)	*N* = 334 respondents aged 18–29, 30–39, 40–49 and ≥ 50 *n* = 55 for the 18–29 subgroup Gender: N/A Race/ethnicity: N/A	Quantitative Secondary data	Category 3 – Cannabis use rates & patterns during the pandemic Characterized trends in daily cannabis use and risk characteristics associated with daily use during the pandemic.	Daily cannabis use was higher among males, young adults aged 18–29, individuals experiencing loneliness, and those very worried about the pandemic’s impact on their finances.
Imtiaz et al. (2021)	Canada (all provinces and territories except Nunavut)	*N* = 3012 adults ≥ 18 Gender: male (53.12%), female (46.88%) Race/ethnicity: non-white (31%), white (68.67%)	Quantitative Secondary data	Category 3 – Cannabis use rates & patterns during the pandemic Characterized trends in cannabis use in the overall population and characterize patterns of and identify risk characteristics associated with an increase among those who use.	Approximately half of the individuals increased their cannabis use compared to the start of the pandemic. Risk characteristics associated with this increase included being in the 18–29 year old age group, having less than a college or university education, and being worried about the financial impact of the pandemic.
Jani et al. (2022)	Ontario	*N* = 9 Black youth aged 16 to 19 years of age Gender: male 78%, 22% female Race/ethnicity: Black, African Caribbean (100%)	Mixed	Category 5 – Prevention tools Explored the feasibility and acceptability of a psychoeducational program on cannabis use and risks of psychosis for Black African Caribbean youth.	The results suggest that this pilot program was acceptable and feasible in addressing the knowledge gap about cannabis and psychosis among underage Black youth, with increased awareness of psychosis and the effects of cannabis.
Kim et al. (2022)	Ontario	*N* = 27,152 Age: youth aged 10–25 presenting to ED Gender: female 2019 (43%), 2020 (41%), 2021 (46%); male 2019 (57%), 2020 (59%), 54%) Race/ethnicity: N/A	Quantitative	Category 2 – Cannabis related injuries & ED visits Investigate the impact of COVID-19 on substance use-related emergency department visits of adolescents and young adults.	Cannabis related ED visits increased in 2020 and in 2021 returned to pre-pandemic level. The rate of ED visits with emergent/life-threatening triage levels (CTAS 1 and 2) significantly increased in 2020 (36.8–38.7%) and remained high in 2021 (38.4%). A significant increase in emergent/life‐threatening triage levels for alcohol‐, opioid‐, cannabis‐ and multiple psychoactive substance‐related ED visits.
Leatherdale et al. (2021)	Ontario & Quebec	*N* = 7567 in 2018, *N* = 8548 in 2019 and *N* = 1937 in 2020 Age = 13–18 years, elementary or high school students (mean age 14) Gender: female (53%) Race/ethnicity: N/A	Quantitative Secondary data	Category 3 – Cannabis use rates & patterns during the pandemic Evaluate the effect of the early stages of the COVID-19 pandemic on youth cannabis use.	There was a greater reduction in the expected increase of daily cannabis use among older male students compared to younger male students. However, there was no reduction in the escalation of daily cannabis use among females of any age group. Notably, 27.3% (*n* = 241) of respondents who reported cannabis use in the past year indicated an increase in their usage due to COVID-19, while 23.2% (*n* = 205) reported a decrease. The largest reduction in the expected escalation of cannabis use was among those who reported less frequent use during pre-COVID periods. Similarly, 27.4% (*n* = 242) of respondents reporting past year cannabis use stated they were using cannabis to cope with COVID-19 related changes.
MacDougall & Maston (2021)	Atlantic Canada	*N* = 20 undergraduate students aged 19–24 from Nova Scotia (*n* = 7), New Brunswick (*n* = 6), Ontario (*n* = 5), and Western Canada (*n* = 2) Note: data were collected in Atlantic Canada, but participants were also from other parts of Canada Gender: N/A Race/ethnicity: N/A	Qualitative	Category 4 – Perceptions related to cannabis use Explored post-secondary students’ perceptions of cannabis use in Atlantic Canada.	Participants perceived an overall increase in cannabis use post-legalization, a reduction in stigma, and a normalization of cannabis in everyday life. The study found a tendency for risk denial among participants. They were aware of some of the risks associated with cannabis use but did not believe these risks applied to their own health. Additionally, they viewed cannabis as healthier than prescription medications. The most commonly acknowledged physical health risk was lung injury. Many participants reported using cannabis to manage mental and physical health issues, including anxiety, sleep problems, stress, and bipolar disorder.
Magier et al. (2022)	Québec, Ontario, Alberta, British Columbia	*N* = 68,037 secondary school students and *N* = 136 school administrators Ages = 13 to 18 years, grades 9–12 or secondary 1–5 in Québec Gender: N/A Race/ethnicity: N/A	Quantitative Secondary data	Category 1 – Prevalence rates, patterns & trends Explored whether different disciplinary approaches for cannabis use are associated with student cannabis use and schools’ support for the prevention and cessation of substance use.	Students who engaged in binge drinking and tobacco smoking were more likely to report current cannabis use. Those with more spending money also reported using cannabis more frequently. None of the disciplinary approaches related to cannabis use were linked to actual use, but they influenced student perceptions. Students who perceived their school as highly unsupportive in prevention and cessation of substance use reported the highest rates of cannabis use. In the year following legalization, the highest rates of cannabis use were in Ontario and Alberta.
Moreno & van Mierlo (2021)	Ontario	*N* = 1110 respondents aged 12–25 Gender: female (40%), male (53%), transgender or nonbinary (5%), N/A (2%) Race/ethnicity: N/A	Quantitative Secondary data	Category 5 – Prevention tools Examined behavioral factors associated with cannabis-impaired driving and explored impact of a digital health intervention aimed at educating youth about responsible use.	Most reported no problems related to their cannabis use (72.7%). However, 48.8% of youth using cannabis daily or almost daily, reported health, social, legal, or financial problems associated with cannabis use. Approximately, 30.8% of youth reported driving after using cannabis, with gender not being a significant factor.
Myran et al. (2023)	Québec Ontario, Alberta, British Columbia	*N* = 581 hospitalizations of children 0–9 years of age (mean age at time of hospitalization was 3.6 years) Gender: male (53.9%), female (46.1%) Race/ethnicity: N/A	Quantitative Secondary data	Category 2 – Cannabis related injuries & ED visits Evaluated changes in proportions of cannabis-related hospitalizations among children (pre- and post-legalization).	Post-legalization, hospitalizations increased to 79%. Provinces allowing edible cannabis sales saw larger increases in unintentional pediatric poisonings and hospitalizations, than those restricting edibles. In provinces with legal and commercialized edibles, about one-third of pediatric poisonings were due to cannabis.
Myran, Pugliese et al. (2022)	Ontario	*N* = 26,581 cannabis attributable ED visits for 15 + age group pre-legalization *N* = 40,545 cannabis attributable ED visits for 15 + post-legalization *n* = 13,490 ED visits in 15–24-year-old youth pre-legalization *n* = 17,542 ED visits in 15–24-year-old youth post-legalization Gender: N/A Race/ethnicity: N/A	Quantitative Secondary data	Category 3 – Cannabis related injuries & ED visits Examined how cannabis retail sales and cannabis-related health harms have changed following legalization through measured changes in cannabis-attributable emergency department (ED) visits.	Cannabis-related ED visits increased after the legalization of cannabis. Commercialization was associated with an increase in rates of cannabis-attributable ED visits affecting the 15-24-year-old age group more than the general 15 + population.
Myran, Roberts et al. (2022)	Ontario	*N* = 8140 ED visits (pre- and post-legalization) by Ontario residents aged 15 and older *n* = 1117 ED visits for 15–18-year-olds *n* = 2834 ED visits for 19-24-year-olds Gender: female (51.1%), male (48.9%) Race/ethnicity: N/A	Quantitative Secondary data	Category 3 – Cannabis related injuries & ED visits Examined changes in the number and characteristics of cannabis hyperemesis syndrome (CHS) emergency department (ED) after legalization of cannabis.	Cannabis increased from the 5th most common codiagnosis with vomiting pre-legalization to the most common during the commercialization period. ED visit rates increased 13-fold from 2014 to 2021. CHS rates were highest in 19–24-year-olds, and attributed to commercialization.
Nguyen et al. (2023)	Canada	*N* = 49,060 youths aged 15–18 years from the CTUMS and *N* = 219,917 students in grades 9–12 from the YSS+. Gender: N/A Race/ethnicity: N/A	Quantitative Secondary data	Category 1 – Prevalence rates, patterns & trends Investigated changes in overall prevalence of youth cannabis use and changes in cannabis initiation among never users after legalization.	Legalization led to higher cannabis initiation rates among never users. Although there was an increase in prevalence rates post-legalization, it was not considered significant.
Nguyen & Mital (2022)	Québec	*N* = 1005 youth aged 15 to 20 years of age Mean age = 17.5 Gender: N/A Race/ethnicity: N/A	Quantitative	Category 1 – Prevalence rates, patterns & trends Investigated changes in youth cannabis use after an increase in minimum legal age for cannabis in Quebec from 18 to 21.	Post-policy implementation, there was an increase in past-3-month cannabis use among 18 to 20 year-olds, but 51% lower in Quebec than in other provinces. No significant change was found among 15 to 17 year-olds.
Pocuca et al. (2022)	Québec	*N* = 1096 individuals aged 21 in pre-pandemic phase and 22 years old during pandemic, 59% were students Gender: female (62%) Race/ethnicity: N/A	Quantitative Secondary data	Category 3 – Cannabis use rates & patterns during the pandemic Examined changes in alcohol and cannabis use among emerging adults from pre-pandemic to during COVID-19.	There were decreases in binge drinking but no changes in overall alcohol and cannabis use.
Robinson et al. (2020)	British Columbia	*N* = 398 students in pre-legalization phase and *N* = 377 students in post-legalization phase aged 13–14 years in elementary or high school Gender: female (50%) pre-legalization, female (46.2%) post-legalization Race/ethnicity: N/A	Quantitative	Category 4 – Perceptions related to cannabis use Examined whether the legalization of recreational cannabis use changed young adolescents’ opinions and perceptions of cannabis.	The most frequent theme was that legalization was perceived by students as having a negative impact.
Romano, Butler et al. (2022)	Québec, Ontario Alberta, British Columbia	*N* = 4763 students in grades 9–12 and secondary 1–5 in Québec who used cannabis in last 12 months. Gender: male (50.2%), female (49.8%). Restricted participation to students who identified gender as male or female Race/ethnicity: N/A	Quantitative Secondary data	Category 1 – Prevalence rates, patterns & trends Explored associations between indicators of risky cannabis use and different modes of cannabis use.	Smoking was the most common cannabis use mode, with one-third using multiple modes. One-third reported using cannabis alone, with more frequent use, and multiple modes of use. Earlier cannabis initiation was linked to multiple modes of use, with few gender differences.
Romano, Patte et al. (2022)	Québec, Ontario Alberta, British Columbia	*N* = 7876 high school or elementary school students Mean age = 15 years Gender: male (36.7%), female (61.4%), Other/prefer not to say (1.9%) Race/ethnicity: white (77.6%), BIPOC (22.4%)	Quantitative Secondary data	Category 2 – Cannabis use rates & patterns during the pandemic Examined how substance use is associated with adolescents’ perceptions of and adherence to early COVID-19 related public health measures.	No link was found between current cannabis use and adherence to COVID-19 restrictions. Youth using cannabis were 34% less likely to view restrictions as too strict, possibly due to the prevalence of solitary cannabis use compared to alcohol.
Salmon et al. (2022)	Manitoba	*N* = 664 respondents aged 16 to 21 years	Quantitative Secondary data	Category 2 – Cannabis use rates & patterns during the pandemic Estimated the associations between a history of Adverse Childhood Experiences (ACEs) and self-reported stressors and symptoms including cannabis use during the pandemic among older adolescents (16–17) and young adults (18–21).	More than half of young adults (52%) and one-third (33%) of older adolescents used cannabis. All ACEs (except physical abuse and spanking) were associated with increased pandemic-related cannabis use among young adults. Among older adolescents, emotional neglect was linked to increased cannabis use. Elevated cannabis use in young adults with ACE histories raised concerns.
Sampasa-Kanyinga et al. (2022)	Ontario	*N* = 7229 students aged 11–20 years Female (49.5%), male (50.5%) Race/ethnicity: Black (9.9%), East/Southeast Asian (14.1%), South Asian (9.1%), other (16.9%), white (49.8%)	Quantitative Secondary data	Category 1 – Prevalence rates, patterns & trends Examined the association between cyberbullying involvement, parental support, and cannabis use among adolescents.	There were no gender differences in cannabis use prevalence (23% past year). Low levels of parental support and cyberbullying (victim or perpetrator) were linked to higher cannabis use odds.
Turuba et al. (2022)	British Columbia	*N* = 30 youth aged 12–24 with lived experience of substance use including cannabis Mean age = 21 years Gender: woman (55.6%), man (33.3%), nonbinary (5.6%), not sure/questioning (5.6%) Race/ethnicity: Caucasian/white (66.7%), Middle Eastern/North African (11.1%), Southeast Asian (11.1%), South Asian (5.6%), Chinese (5.6%), First Nation/Métis/Inuit (5.6%)	Qualitative	Category 4 – Perceptions related to cannabis use Explored how youth perceive and experience substance use services in British Columbia.	Cannabis was the second most used substance after alcohol. Youth believed service providers often overlooked cannabis use and its addictive nature. Youth felt this invalidated their experiences. Youth sought more information on addictions and better access to substance use services.
Vignault et al. (2021)	Québec	Underage population (12–17 years), *n* = 41 (prelegalization), *n* = 45 (postlegalization). Adult population (18 and over), *n* = 969 (prelegalization), *n* = 1002 postlegalization) Gender: N/A Race/ethnicity: N/A	Quantitative Secondary data	Category 1 – Prevalence rates, patterns & trends Examined the impact of the legalization on active cannabis use, cannabis use disorder, and psychotic disorders.	Among 12-17-year-olds, cannabis use increased from 17.9% pre-legalization to 25.5% post-legalization. Substance use disorders (SUDs) involving cannabis increased from 4.8–12.8%, and psychotic disorder diagnoses from 2.4–6.4%. These were not statistically significant differences due to the small sample size. In adults, significant differences were noted in the 18–24-year-old age group post-legalization: 52.3% used cannabis (up from 37.9% pre-legalization), and cannabis use disorder increased from 17.3–25.9% post-legalization. There were also increased psychiatric visits for this age group.
Williams et al. (2021)	Québec, Ontario Alberta, British Columbia	*N* = 738 students in grades 9 & 10 or secondary 3 in Québec who participated in 3 waves of the study. Gender: female 53%, male 47% Race/ethnicity: white (73%), non-white (27%)	Quantitative Secondary data	Category 1 – Prevalence rates, patterns & trends Examined associations between longitudinal patterns of substance use including cannabis use and anxiety and depression symptoms among secondary school students.	There were three use classes: occasional alcohol and ecigarette use (50%), escalating polysubstance use (25%), and consistent polysubstance use (25%), including weekly cannabis use. Consistent polysubstance use was linked to depression but not anxiety, and escalating use to depression for males.
Yang et al. (2022)	Québec, Ontario Alberta, British Columbia	*N* = 9307 students in grades 9 & 10 including secondary 1–5 in Québec. Gender: N/A Race/ethnicity: Black 3%, Asian 6.8%, Latin American 2.1%, white 73.8%, other 13.8%, missing 0.5%	Quantitative Secondary data	Category 1 – Prevalence rates, patterns & trends Explored the transitions of polysubstance use patterns across time among youth.	The prevalence of “never use” decreased over time, while occasional and current use of cannabis increased across all three waves. Cannabis use prevalence was 4.0% in 2016-17, 9.5% in 2017-18, and 16.1% (2018-19).
Yeung et al. (2021)	Alberta	*N* = 1920 children & youth 0–17 years of age pre-legalization *N* = 602 children & youth aged 0–17 post-legalization Age = Subgroups included children (aged 0–11 years), younger adolescents (12 to 14 years), and older adolescents (15 to 17 years) presenting to ER Gender: N/A Race/ethnicity: N/A	Quantitative Secondary data	Category 2 – Cannabis related injuries & ED visits Examined changes in cannabis-related pediatric emergency department visits pre- and post-legalization.	Pre-legalization ED visits included 2.7% children < 12 years, 17.5% aged 12–14, and 79.9% aged 15–17. Post-legalization, increases in children < 12 (6.6%), and 12-14-year-olds (20.4%) occurred, with a decrease in 15–17 year-olds (72.9%). Hyperemesis cases increased in older adolescents aged 15–17. Overall, pediatric cannabis-related ED visits did not change, but the rate and proportion of children < 12 increased, primarily due to a 77% post legalization increase in unintentional ingestion.
Yousufzai et al. (2022)	Canada	*N* = 312 young adults 18–29 years old (mean age = 22.79) recruited through Ontario Tech University and social media Gender: female (61%), male (39%) Race/ethnicity: nonwhite/mixed (42%), white (57%)	Quantitative	Category 3 – Cannabis use rates & patterns during the pandemic Examined changes in self-reported frequency of inhaled routes of cannabis consumption post-legalization, the EVALI epidemic, and COVID-19 pandemic.	Between 2018 and 2020, there was an increase in smoking and vaping of cannabis, with males reporting higher frequencies than females. The most notable increase was post-legalization.
Zhang et al. (2022)	Ontario	*N* = 1,079 pediatric cases Female (60%)	Quantitative Secondary data	Category 2 – Cannabis related injuries & ED visits Examined whether there was an increase in unintentional poisoning, recreational drug use, and intentional self-harm poisoning presentations in children under 18 during the COVID-19 pandemic.	Despite a 42.4% decrease in total ED visits during the pandemic, unintentional, recreational, and intentional drug exposures more than doubled. The proportion of cannabis cases increased from 28–40.4% with teens showing the greatest increase by a third. Teen poisonings were mainly due to intentional self-harm and recreational drug use, consistent across cannabis, vaping, other recreational drugs, and multisubstance drug use during the pandemic.
Zuckermann Battista et al. (2021)	Québec, Ontario Alberta, British Columbia	Cross-sectional sample: *N* = 102,685 youth Longitudinal sample: *N* = 5400 youth Samples included youth in grades 9–12 or secondary 3–5 in Québec.	Quantitative Secondary data	Category 1 – Prevalence rates, patterns & trends Investigate the effects of cannabis legalization on youth cannabis use rates.	Cannabis use was significantly increased after legalization. Provincial differences showed lower regular use rates in Québec and British Columbia.
Zuckermann Gohari et al. (2021)	Québec, Ontario Alberta, British Columbia	*N* = 2953 secondary school students in grades 9–12. Gender: N/A Race/ethnicity: N/A	Quantitative Secondary data	Category 1 – Prevalence rates, patterns & trends Investigated the factors associated with changes in cannabis use modes among youth before and after legalization.	Multimodal cannabis use among youth increased. Over 42% of youth shifted to multiple modes versus 31.3% who maintained a single mode. 14.3% maintained multiple modes and only 12.1% reduced. Students maintaining multiple modes were more likely to have high weekly spending money, to binge drink or vape, to use cannabis regularly, and to report more depression symptoms.
Zutrauen et al. (2022)	Canada (no specification of province)	*N* = 71 cases of children 17 years of age and younger Gender: female (43.7%), male (56.3%) Race/ethnicity: N/A	Quantitative Secondary data	Category 2 – Cannabis related injuries & ED visits Investigated acute vaping-related injury/illness cases associated with the inhalation of vaping aerosols among children and adolescents presenting to Canadian pediatricians.	68% of patients were 15 to 17 years of age. Cannabis was the cause in 24% of vaping-related injuries, with 24% of these being severe.

### Findings of selected studies

**Category 1: Prevalence, patterns, trends and characteristics (n = 17)**: Among the seven studies examining cannabis use rates, 71.42% found that the prevalence has increased post-legalization, especially among young adults aged 18–24 [4, 15, 56, 69, 76]. For example, Fischer et al. [56] examined data from three surveys and found that rates were highest among young adults in all three surveys, with significant increases after legalization. The study also found that 40% of respondents under the legal age reported it was very easy to access cannabis. Another study by Vignault et al. [15] found increased use of cannabis and an increase in the diagnosis of cannabis use disorder, with more significant increases in young adults aged 18–24. Hammond et al. [76] examined data of 16–19 year olds in Canada (n = 11,779), United States (n = 11,689), and England (n = 11,117), finding that the prevalence was higher in Canada and the United States, and prevalence of daily use, past month, and past 12 months increased from 22.5% in 2017, to 23.4% in 2018, and to 27% in 2019. Zuckermann, Battista et al. [69] examined the prevalence of use in high school students and found that the prevalence of ever use were significantly higher the year following legalization, but rates of regular use stayed the same. The study also found provincial differences with lower rates in Québec and British Columbia. In contrast, Gueye et al. [88] found that cannabis use levels only increased for young adults aged 18–24 who were using regularly, defined as three times or more in the past month. Nguyen & Mital [4] examined changes in cannabis use among youth and young adults aged 15–20 in Québec before and after the province increased the minimum legal age (MLA) for cannabis use from 18 to 21 years. The study found that the MLA change was not associated with changes in cannabis use among youth aged 15 to 17. However there was an increase in cannabis use among those aged 18 to 20. Notably, this increase was 51% lower in Québec compared to other provinces, a difference that was attributed to the increase in the MLA. Contrary to the other studies in the subgroup examining prevalence rates, the study by Nguyen et al. [60], which focused on cannabis use in 15-18-year-olds, did not find a significant increase in prevalence rates. However, it did report increases in the initiation of cannabis use among never users and easier access to cannabis.

Among the ten studies examining patterns and trends in youth and young adult cannabis use, several findings stand out as particularly noteworthy. Firstly, only a few studies examined or found gender differences in patterns or modes of use [65, 67, 75]. Hammond et al. [75] and Williams et al. [67] found a higher likelihood of daily or weekly cannabis use among males, while Romano, Butler et al. [65] did not find a significant gender difference in risky cannabis use behaviours and mode of use. An increase in multimodal cannabis use was another trend identified by some studies [65, 70, 75], although smoking remains the most common mode of use [65]. Studies found that multiple modes of cannabis use were associated with higher frequency of use and solitary use [65, 79], as well as depressive symptoms [70]. Hammond et al. [75] found that the standard size of a joint increased from 0.2 g in 2018 to 1.0 g in 2020. Three studies found that polysubstance use was pervasive among youth and young adults [67, 68, 79]. Yang et al. [68] identified four distinct polysubstance use patterns in middle and high school students: (1) no use, (2) single use of alcohol, (3) dual use of cigarettes and alcohol, and (4) multi-use. They found that youth are more likely to remain in the same subgroup of use pattern or transition to a higher use group as they age. The study also showed that cannabis use rates were four times higher in 2018-19 than they were in 2016-17. Williams et al. [67] found that consistent polysubstance use was associated with depression in high school students.

Two studies examined youth cannabis use rates and school approaches [63, 95]. Butler et al. [95] found no significant changes in school priorities pre- and post-legalization. Substance use was not a leading priority, and school prioritization of cannabis use was not associated with student cannabis behaviours. Magier et al. [63] found that school disciplinary approaches were not associated with cannabis use, but they were associated with student perceptions. Students who perceived their school to be more unsupportive reported higher rates of cannabis use. Finally, a study by Hammami & Katapally [92] with a sample of 818 youth found that cannabis use was a risk factor associated with a higher suicide ideation risk for youth aged 13–18.

**Category 2: Cannabis-related injuries and ED visits (n = 12)**: Most studies in the second category showed an increase in ED visits since legalization [64, 74, 77, 81, 83, 84, 86, 87, 93] with two focused on unintentional cannabis-related injuries or poisonings [64, 77], one on intentional injuries [74], and the remaining studies reporting on both unintentional and intentional cannabis-related injuries [81, 83, 84, 86, 87, 93]. One study on unintentional exposures found that 76% of accidental exposures were attributed to edible products [77]. Another study compared unintentional exposures in the pre-legalization and post-legalization data. They found that of the 581 hospitalizations for cannabis poisoning, 20.65% were pre-legalization and 79.35% occurred post-legalization [64]. An Alberta study also found an increase in unintentional ingestions in children 0–11, as well as adolescents aged 15–17 [93]. A Québec study found increases in hospitalizations post-legalization among boys aged 10–14 years, with 70% of substance-related hospitalizations after legalization involving cannabis, compared with 39.3% pre-legalization [87]. Kim et al. [81] studied ED visits in Ontario and found that cannabis-related visits increased in 2020 compared to 2019 among young adults 18–25 years more than among youth 10–17 years. Additionally, young adults with cannabis-related ED visits had triage to end times longer than six hours with higher rates of emergent or life threatening triage levels. Another Ontario study found that there was an increase in cannabis poisonings due to recreational use among 16-18-year-olds. The proportion of cannabis cases increased from 28% in 2018 to 40.4% in 2020 among those aged 16–18, with no significant changes in poisonings from other substances including alcohol, opioids, acetaminaphen, and prescription medications [86].

Three studies reported on increased cases of cannabinoid hyperemesis syndrome (CHS) post-legalization, a condition more commonly observed in individuals with cannabis use disorder or those who use cannabis chronically [74, 84, 93]. Andrews et al. [74] found that the treatment prevalence for CHS doubled between 2017 and 2021 among youth and young adults aged 16–24 in Alberta and also reported that youth and young adults with CHS had high rates of co-occurring anxiety and depression.

Two studies examined vaping-related injuries (VAPI), with one focused on VAPI patients aged 15 to 50+ [53] and the other examining VAPI cases aged 17 years and younger [62]. Zutrauen et al. [62] found that that 68% of patients were 15–17 years old and nicotine vaping was responsible for 42% of injuries, with cannabis accounting for 24% of vaping-related injuries. Baker et al. [53] reported that 25% of vaping-associated lung illness (VALI) patients were youth and young adults aged 16–19 and 40% report vaping or dabbing cannabis. The authors noted that 80% of VALI patients were hospitalized. Data for young adults aged 20–24 is unavailable because they were categorized with the 25–49 year old age group.

Three studies attributed increased pediatric poisonings, ED visits, and hospitalizations to the commercialization of cannabis [64, 83, 84]. Myran, Pugliese et al. [83] found that there were significant increases in cannabis-related ED visits, with the most changes in youth and young adults aged 15–24. The authors noted that this coincided with large increases in the number of cannabis stores and sales. All three studies on cannabis-related injuries and commercialization recommend stricter regulations on the number of cannabis dispensaries, the sale of products appealing to youth and young adults such as edibles, and the minimum legal age for purchasing cannabis [64, 83, 84]. One study, differing in focus from others examining ED visits, was by Callaghan, Sanches et al. [72]. This study examined traffic-injury ED presentations since the legalization of cannabis and found no significant changes.

**Category 3: cannabis use rates and patterns during the COVID-19 pandemic (n = 9)**: In this category, most studies examining youth and young adult cannabis use rates during the pandemic found that the rates increased, and isolation was associated with increased rates [17, 61, 78]. Three studies examining trends found that the odds of increased cannabis use were higher for those ages 18–29 [58, 59] and for youth who were using cannabis to cope with the pandemic [71]. A study by Pocuca et al. [20] during the first three months of the pandemic in Québec found that overall there were no changes in cannabis use, but noted that young adults with preexisting vulnerabilities increased their substance use. Another study examining COVID-19 measures and substance use did not find a relationship between adherence to these measures and cannabis use. The study reported that youth using cannabis perceived these measures as less strict, which was interpreted to be related to the perception that alcohol use is more social, while cannabis use is more solitary [66]. Salmon et al. [89] examined characteristics and trends of cannabis use in youth and young adults with adverse childhood experiences (ACEs) during the COVID-19 pandemic. The study found that youth and young adults with a history of ACEs experienced increased pandemic-related stressors and showed increased use of alcohol and cannabis.

**Category 4: Perceptions related to cannabis use (n = 6)**: Robinson et al. [90] examined the differences in perceptions of grade 8 students regarding the legalization of cannabis, both pre- and post-legalization. The study found that adolescents generally held negative views toward cannabis legalization, and these perspectives did not change post-legalization. Harris-Lane et al. [57] found that young adults aged 18–25 years tend to minimize their cannabis use and are more adept at perceiving risks in young adolescents than in themselves. In a similar vein, MacDougall & Maston [73] observed that undergraduates in their study reported an overall reduction in stigma post-legalization, and a normalization of cannabis in everyday life. The authors noted a “risk denial” among participants who described cannabis as healthier than prescription medication and used it to manage physical and mental health problems. Another study conducted in Newfoundland & Labrador indicated that youth and young adults do not believe that they receive adequate education about cannabis across the province and expressed a need for more evidence-informed cannabis education through media and schools [Bishop et al., 2022]. Turuba et al. [91] explored how youth and young adults perceive substance use services in British Columbia. Participants highlighted that cannabis use is often overlooked by service providers, noting a lack of recognition that cannabis is an addictive substance. They emphasized the need for more information about addictions and noted that there are challenges in accessing substance use services. The final study in this category examined perceptions of cannabis-related penalties for immigrants and found that right-wing ideology was associated with more support for harsher penalties for immigrants than non-immigrants [54].

**Category 5: Prevention tools for youth and young adults (n = 2)**: Jani et al. [80] evaluated an online psychoeducational program for Black youth and young adults on cannabis and psychosis with nine participants aged 16–19. Participants showed increased awareness after playing the psychoeducational video game, which was supplemented with a tutorial intervention [80]. The second study examined how a digital tool can provide information about responsible cannabis use and prevent cannabis-impaired driving. The study found that 73.6% of youth and young adult respondents used cannabis, with 30.8% reporting that they drove after cannabis use. Additionally, gender was not found to be a significant determinant of driving after cannabis use [82].

**Category 6: Cannabis-related offences (n = 1)**: There was only one study in this category, which examined police-reported cannabis-related crime among youth aged 12–17 [55]. The study found decreases in cannabis-related crimes among male and female youth in this age group. This finding followed a decreasing trend in police reported cannabis-related incidents pre-legalization, attributed to normalization and decriminalization. The study reported a 65% decrease in cannabis-related crimes for females and 55% decrease among males over 76 days post-legalization. However, the study was limited to a short-term period and did not disaggregate the data by race or ethnicity, which is necessary to determine if the decreases occurred among populations historically overrepresented in the criminal justice system.

## Discussion

In this scoping review, we identified and synthesized 47 studies that examined youth and young adult cannabis use in Canada since its legalization. The review aimed to examine the extent, nature, and range of available evidence on this topic to inform and enhance policies, services, and public education strategies. Several key findings emerged from the review. First, regarding prevalence, most studies indicated an increase in cannabis use and cannabis use disorders among young adults aged 18–24 since legalization. During the pandemic, higher rates of use were reported, particularly among young adults aged 18–29 years and those who had never used cannabis before the pandemic. For youth under 18, the results were mixed: some studies reported increased overall rates, others found no significant changes, and a few noted earlier initiation rates. There was also an increased likelihood of higher usage rates among youth post-legalization, especially in those who had already been using cannabis frequently before legalization. In terms of trends, the studies showed that smoking is the most common mode of use, but multimodal and polysubstance use are increasingly observed among youth and young adults. Most studies that examined ED visits and hospitalizations due to cannabis-related injuries reported an increase in visits for both intentional and unintentional poisonings in young children and teens.

Since legalization, the increased prevalence of cannabis use among youth and young adults, as well as the increase in cannabis-related injuries and emergency department visits, can be attributed to several interconnected factors. Notably, the enhanced availability and accessibility of cannabis is a contributing factor. Manthey et al. [30] conducted a systematic review on the impact of legal cannabis availability and found that higher availability was associated with an increased likelihood of cannabis use. The review also identified a link between the number of cannabis retail stores and youth cannabis use, despite legal age restrictions on cannabis purchases. The authors offer several explanations for this trend, including the lack of strict enforcement of age restrictions at cannabis outlets and youths’ access to cannabis through family and friends. Furthermore, they suggest that high availability and less restrictive regulatory measures contribute to the normalization of cannabis use, leading to higher rates of experimentation and increased demand. This explanation has been supported by studies on trends in Oregon [96] and Uruguay [97] which showed that youth found it easier to obtain cannabis post-legalization. Enforcing age restrictions may have some challenges in Canada, with 38.2% of young people aged 16–19 reporting that they have purchased cannabis from a legal storefront and 3.9% from a legal online store [2].

Research suggests that the commercialization of cannabis plays a role in its high prevalence of use among youth and young adults [34]. Myran et al. [34] conducted a scoping review on the health harms associated with cannabis commercialization and reported mixed results, with five studies out of 14 indicating increases in health harms post-legalization. The authors explain that these mixed findings are related to the level of market maturity, noting that most studies focused on changes within the year following legalization. They argue that cannabis-related harms may be more accurately assessed as the market (e.g., the expansion of retail stores) and products (e.g., those with higher THC levels) grow. One of the studies selected for this review observed higher rates of cannabis-related hospitalizations in three provinces (Ontario, Alberta, British Columbia) that allow the sale of edibles, in contrast to Québec, which prohibits the sale of edibles appealing to children, including sweets, desserts, chocolates and candies. During the examined period, rates of cannabis-related hospitalizations more than doubled in the three provinces that permitted the sale of commercial edibles, while in Québec the rate of hospitalizations remained unchanged [64].

A study examining access to legal cannabis stores since legalization, found a 122.3% increase in per capita stores, with considerable differences between jurisdictions and between public and private retail models. For example, Ontario’s private retail model offers extensive access with 1,552 cannabis stores, in contrast to Québec, which has 91 stores under a public model [64] managed by the Société Québécoise du cannabis (SQDC). This public model mandates that most profits be directed to research and prevention strategies [99].

Some studies in our review suggest that the high prevalence of cannabis use among youth and young adults can be partly attributed to decreased perceptions of its risks and harms. This trend of minimizing of risks is also reflected in responses to the Canadian Cannabis Survey, which found that only 14.8% of individuals over 16 who regularly smoke cannabis perceive it as a serious risk, compared to 75.7% of respondents who regularly smoked tobacco in the past 12 months [2]. Ali et al. [100] highlight how public discourse influences the initiation and continued use of substances among youth. They discuss the normalization and social acceptability of substances like cannabis, along with increased availability, and how these factors may affect usage among youth and young adults. This study draws on the normalization framework introduced by Parker and colleagues [101], which outlines shifts in cultural attitudes and behaviours resulting in a substance being less stigmatized. Using this framework, Asbridge et al. [102] compared the normalization of cannabis with the denormalization of tobacco. Denormalization refers to the adoption new values and rejection of certain behaviours that were viewed as acceptable or mainstream. In the case of tobacco, policies and educational efforts have played a crucial role in altering social and cultural norms. The study emphasized that perceptions of health risks significantly influence the processes of normalization and denormalization, suggesting that while normalization can reduce stigma, it must be balanced with education on harms and risks [100].

Public awareness of the risks and harms associated with cannabis use among youth and young adults remains relatively low. Enhancing public awareness of these health risks is an objective in the Cannabis Act [3], yet 48% of Canadians report not having noticed any educational campaigns or public health messages about cannabis. Furthermore, 52% have not observed health warning messages on cannabis products, and only 11% of Canadians have noticed public health messages about cannabis in health care settings [2]. The Canadian Centre on Substance Use and Addiction (CCSA) [103] conducted a public consultation after five years of cannabis legalization and found persistent misinformation and low cannabis literacy among Canadians. They indicated that this limited understanding of risks and harms suggests Canadians may not be making informed health choices. The CCSA recommended more robust public education efforts, especially aimed at priority groups such as youth. There are also knowledge and skills gaps in service providers, highlighting the need for increased training and education programs focused on youth and young adult cannabis use [104].

The evidence gathered from this scoping review underlines the need to enhance efforts to to protect youth and young adults and minimize risks and harms associated with cannabis legalization. In 2016, the Government of Canada formed a Task Force in preparation for legalization, responsible for developing a framework for the legalization and regulation of cannabis [41]. The Task Force highlighted the risks of cannabis use, particularly to children, youth, young adults, and other vulnerable groups. To mitigate these risks, it recommended a public health approach that “delays the age of initiation, reduces the frequency of use, reduces higher-risk use, reduces problematic use and dependence, expands access to treatment and prevention programs, and ensures early and sustained public education and awareness” [41p15]. Enphasizing the ongoing brain development in young adults aged 18–25, the Task Force stressed the importance of public health measures to discourage and delay cannabis use, including through advertising restrictions and public education. In 2022, an Expert Panel was formed to conduct a Legislative Review of the Cannabis Act [105]. The Expert Panel wrote a report published in March 2024 which emphasized the need for increased efforts to reduce cannabis use in youth and young adults and highlighted the importance of increasing public awareness about the health risks associated with cannabis use through prevention initiatives. The panel agreed with the pre-legalization Task Force, which stated that “revenue generation should be a secondary consideration for all governments, with the protection and promotion of public health and safety as the primary goals” [105p19].

The need to strengthen the public health approach was further emphasized in a recent systematic review that examined how the literature describes and defines a public health approach to substance use [40]. The review argued that an effective public health approach in jurisdictions with legalized cannabis should include the best available evidence, a focus on reducing harm by addressing modifiable risk factors, efforts to minimize commercialization, and regulation measures such as availability, marketing, and promotion of cannabis [40].

Our findings identified several gaps in the current research on cannabis use in youth and young adults. First, there is a notable scarcity of qualitative or mixed-method studies, with a high percentage of research relying on secondary and aggregate data. The scarcity of qualitative data, along with the abundance of secondary data, limits our understanding of how cannabis use affects young people. Additionally, few studies examined the social determinants of health in relation to cannabis use. Despite sexual and gender minority youth (SGMY) in Canada having the highest rates of cannabis use, none of the studies in this review collected data on sexual orientation. Furthermore, many studies did not include racial data, and in instances where race was presented, it was often categorized as white or *non-white*. Also, the studies frequently presented gender in a binary manner (male or female) or categorized gender as male, female, and *other*.

Another literature gap is the tendency to group young adults aged 18–24 with the general adult population. There is ample evidence of increased cannabis use among young adults following legalization, suggesting that they should be treated as a distinct group in future studies. Only two studies explored digital prevention programs, and none focused exclusively on cannabis treatments or interventions. Research on the relationship between cannabis use and physical and mental health was also limited.

There was only one study examining the impact of legalization on cannabis-related offenses among youth (12–17), which showed short-term reductions in criminalization [55]. Since this search was conducted, another study was published, examining cannabis-related criminalization among youth and identifying significant reductions in cannabis-related offenses. These findings show important progress in deterring illicit activities, which is also one of the objectives of the Cannabis Act [106]. Nonetheless, there is a need for future studies to collect and present disaggregated data by race and ethnicity to determine whether there have been reductions in cannabis-related criminalization among racialized youth who have historically faced unjust and disproportionate persecution for cannabis possession and use [107].

## Strengths and limitations

One of the inherent limitations of a scoping review is the lack of quality appraisal of the studies, although this is not its primary objective. Instead, a scoping review aims to map out the literature and provide a broader perspective on the topic. We excluded grey literature due to feasibility issues;, it is harder to locate, often lacks abstracts for screening, and varies widely in quality due to the absence of peer review and journal standards. Another potential limitation of this review is the unintentional omission of relevant studies. We minimized this risk by ensuring our research team includes a librarian with expertise in relevant databases, who was also responsible for developing a robust search strategy. We also conducted three searches to try to capure the most recent literature since the last search. Our scoping review had many strengths including being the first to synthesize studies focused on youth and young adult cannabis use since legalization in Canada. We adhered to the PRISMA-ScR checklist, which increased transparency and reduced bias. Finally, two screeners independently revieweds each article, with a third screener resolving any discrepancies.

## Conclusions

Recreational cannabis was legalized in Canada in 2018, raising concerns about its prevalence among youth and young adults, along with the associated risks and harms. Maintaining a public health approach is critical, focusing on reducing the high prevalence of cannabis use among youth and young adults. This involves implementing prevention strategies to minimize harms, enhancing public education, reducing commercialization, limiting youth access to cannabis, promoting guidelines for lower-risk cannabis use, and increasing training for healthcare providers. While this scoping review addressed some gaps in the literature, it also highlights the existence of many remaining knowledge gaps. There is a pressing need for further research to inform policy, practice, education, and training.

### Electronic supplementary material

Below is the link to the electronic supplementary material.

Supplementary Material 1

Supplementary Material 2

## Data Availability

No datasets were generated or analysed during the current study.

## Abbreviations

ACEs: Adverse Childhood Experiences
CHS: Cannabinoid Hyperemesis Syndrome
CCSA: Canadian Centre on Substance Use and Addiction
CPS: Canadian Pediatric Society
DALY: Disability-Adjusted Life Year
ED: Emergency Department
MLA: Minimum Legal Age
OSDUHS: Ontario Student Drug and Health Survey
PRESS: Peer Review of Electronic Search Strategies
PRISMA-ScR: PRISMA Extension for Scoping Reviews
SGMY: Sexual and Gender Minority Youth
SQDC: Société Québécoise Du Cannabis
THC: Tetrahydrocannabinol
VALI: Vaping-Associated Lung Illness
VAPI: Vaping-Related Injuries
YLD: Years of Healthy Life Lost due to Disability

## References

[1] United Nations Office on Drugs and Crimes. World Drug Report: Cannabis and hallucinogens 2022. https://www.unodc.org/unodc/en/data-and-analysis/world-drug-report-2022.html.

[2] Health Canada. Canadian Cannabis Survey 2022 [Internet]. https://www.canada.ca/en/health-canada/services/drugs-medication/cannabis/research-data/canadian-cannabis-survey-2022-summary.html.

[3] Government of Canada. Controlled Drugs and Substances Act. Food and Drugs Act. Cannabis Act (Cannabis Regulations SOR/2018 – 144). Justice Laws. 2018. https://www.canada.ca/en/health-canada/services/drugs-medication/cannabis/research-data/canadian-cannabis-survey-2022-summary.html.

[4] Nguyen HV, Mital S (2022). Changes in youth cannabis use after an increase in cannabis minimum legal age in Quebec, Canada. JAMA Netw Open.

[5] Wadsworth E, Driezen P, Chan G, Hall W, Hammond D (2022). Perceived access to cannabis and ease of purchasing cannabis in retail stores in Canada immediately before and one year after legalization. Am J Drug Alcohol Abuse.

[6] Boak A, Elton-Marshall T, Hamilton HA. The well-being of Ontario students: findings from the 2021 Ontario Student Drug Use and Health Survey (OSDUHS). 2022. https://youthrex.com/report/the-well-being-of-ontario-students-findings-from-the-2021-ontario-student-drug-use-and-health-survey/.

[7] Butler A, Patte KA, Ferro MA, Leatherdale ST (2019). Interrelationships among depression, anxiety, flourishing, and cannabis use in youth. Addict Behav.

[8] Sorkhou M, Bedder RH, George TP (2021). The behavioral sequelae of cannabis use in healthy people: a systematic review. Front Psychiatry.

[9] Fischer B, Hall W, Fidalgo TM, Hoch E, Foll BL, Medina-Mora ME, Reimer J, Tibbo PG, Jutras-Aswad D (2023). Recommendations for reducing the risk of cannabis use-related adverse psychosis outcomes: a public mental health-oriented evidence review. J Dual Diagn.

[10] Godin SL, Shehata S (2022). Adolescent cannabis use and later development of schizophrenia: an updated systematic review of longitudinal studies. J Clin Psychol.

[11] Hosseini S, Oremus M (2019). The effect of age of initiation of cannabis use on psychosis, depression, and anxiety among youth under 25 years. Can J Psychiatry.

[12] Maloney-Hall B, Wallingford SC, Konefal S, Young MM (2020). Original quantitative research-psychotic disorder and cannabis use: Canadian hospitalization trends, 2006–2015. Health Promot Chronic Dis Prev Can.

[13] Polkosnik GL, Sorkhou M, George TP (2021). Effects of cannabis use on psychotic and mood symptoms: a systematic review. Can J Addict.

[14] Santesteban-Echarri, O., Liu, L., Miller, M., Bearden, C. E., Cadenhead, K. S., Cannon,T. D., … Addington, J. (2022). Cannabis use and attenuated positive and negative symptoms in youth at clinical high risk for psychosis. Schizophr Res. 2022;248:114–21. 10.1016/j.schres.2022.08.005 36030758

[15] Vignault C, Massé A, Gouron D, Quintin J, Asli KD, Semaan W. (2021). The potential impact of recreational cannabis legalization on the prevalence of cannabis use disorder and psychotic disorders: A retrospective observational study. Can J Psychiatry, 2021;66:1069–76. 10.1177/0706743720984684 PMC8689454 33567893

[16] Xue S, Husain MI, Zhao H, Ravindran AV (2021). Cannabis Use and Prospective Long-Term Association with anxiety: a systematic review and Meta-analysis of longitudinal studies: usage du cannabis et association prospective à long terme avec l’anxiété: une revue systématique et une méta-analyse d’études longitudinales. Can J Psychiatry.

[17] Bartel SJ, Sherry SB, Stewart SH (2020). Self-isolation: a significant contributor to cannabis use during the COVID-19 pandemic. Subst Abus.

[18] Langlois C, Potvin S, Khullar A, Tourjman SV (2021). Down and high: reflections regarding depression and cannabis. Front Psychiatry.

[19] Harrell MB, Clendennen SL, Sumbe A, Case KR, Mantey DS, Swan S (2022). Cannabis vaping among youth and young adults: a scoping review. Current addiction reports. Curr Addict Rep.

[20] Pocuca N, London-Nadeau K, Geoffroy MC, Chadi N, Séguin JR, Parent S, Boivin M, Tremblay RE, Côté SM, Castellanos-Ryan N. Changes in emerging adults’ alcohol and cannabis use from before to during the COVID-19 pandemic: evidence from a prospective birth cohort. Psychol Addict Behav. 2022. 10.1037/adb0000826 35201807

[21] Zuckermann AM, Williams GC, Battista K, Jiang Y, de Groh M, Leatherdale ST (2020). Prevalence and correlates of youth poly-substance use in the COMPASS study. Addict Behav.

[22] Leos-Toro C, Fong GT, Meyer SB, Hammond D (2020). Cannabis health knowledge and risk perceptions among Canadian youth and young adults. Harm Reduct J.

[23] Carvalho AF, Stubbs B, Vancampfort D, Kloiber S, Maes M, Firth J, Kurdyak PA, Stein DJ, Rehm J, Koyanagi A (2019). Cannabis use and suicide attempts among 86,254 adolescents aged 12–15 years from 21 low-and middle-income countries. Eur Psychiatry.

[24] Behrendt S, Beesdo-Baum K, Höfler M, Perkonigg A, Bühringer G, Lieb R, Wittchen HU (2012). The relevance of age at first alcohol and nicotine use for initiation of cannabis use and progression to cannabis use disorders. Drug Alcohol Depend.

[25] Grant CN, Bélanger RE. Cannabis and Canada’s children and youth. Paediatrics & child health. 2017;22(2):98–102. https://cps.ca/en/documents/position/cannabis-children-and-youth. 10.1093/pch/pxx017 PMC5804770 29480902

[26] Leung J, Chan GC, Hides L, Hall WD (2020). What is the prevalence and risk of cannabis use disorders among people who use cannabis? A systematic review and meta-analysis. Addict Behav.

[27] Mental Health Commission of Canada (MHCC). Cannabis and Mental Health: Priorities for Research in Canada [Internet]. Ottawa (CA): Mental Health Commission of Canada. 2019. https://www.mentalhealthcommission.ca/wp-content/uploads/drupal/2019-07/Cannabis_mental_Health_Summary_july_2019_eng.pdf.

[28] Knapp AA, Lee DC, Borodovsky JT, Auty SG, Gabrielli J, Budney AJ (2019). Emerging trends in cannabis administration among adolescent cannabis users. J Adolesc Health.

[29] Lim CC, Sun T, Leung J, Chung JY, Gartner C, Connor J, Hall W, Chiu V, Stjepanović D, Chan GC (2022). Prevalence of adolescent cannabis vaping: a systematic review and meta-analysis of US and Canadian studies. JAMA Pediatr.

[30] Manthey J, Jacobsen B, Hayer T, Kalke J, López-Pelayo H, Pons-Cabrera MT, Verthein U, Rosenkranz M (2023). The impact of legal cannabis availability on cannabis use and health outcomes: a systematic review. Int J Drug Policy.

[31] O’Grady MA, Iverson MG, Suleiman AO, Rhee TG. Is legalization of recreational cannabis associated with levels of use and cannabis use disorder among youth in the United States? A rapid systematic review. Eur Child Adolesc Psychiatry. 2022 May;4:1–23. 10.1007/s00787-022-01994-9 35508822

[32] Wadsworth E, Craft S, Calder R, Hammond D (2022). Prevalence and use of cannabis products and routes of administration among youth and young adults in Canada and the United States: a systematic review. Addict Behav.

[33] Hall W, Stjepanović D, Dawson D, Leung J (2023). The implementation and public health impacts of cannabis legalization in Canada: a systematic review. Addiction.

[34] Myran DT, Imtiaz S, Konikoff L, Douglas L, Elton-Marshall T (2023). Changes in health harms due to cannabis following legalisation of non‐medical cannabis in Canada in context of cannabis commercialisation: a scoping review. Drug Alcohol Rev.

[35] Alvarez L, Colonna R, Kim S, Chen C, Chippure K, Grewal J, Kimm C, Randell T, Leung V (2021). Young and under the influence: a systematic literature review of the impact of cannabis on the driving performance of youth. Accid Anal Prev.

[36] Lorenzetti V, Hoch E, Hall W (2020). Adolescent cannabis use, cognition, brain health and educational outcomes: a review of the evidence. Eur Neuropsychopharmacol.

[37] Lanthier-Labonté S, Dufour M, Milot DM, Loslier J (2020). Is problematic internet use associated with alcohol and cannabis use among youth? A systematic review. Addict Behav.

[38] Petros R, Walker DD, Pierce A, Monroe-DeVita M (2023). Scoping review of cannabis-reduction psychosocial interventions and reasons for use among young adults with psychosis. J Dual Diagnosis.

[39] Rubin-Kahana DS, Crépault JF, Matheson J, Le Foll B (2022). The impact of cannabis legalization for recreational purposes on youth: a narrative review of the Canadian experience. Front Psychiatry.

[40] Crépault JF, Russell C, Watson TM, Strike C, Bonato S, Rehm J (2023). What is a public health approach to substance use? A qualitative systematic review and thematic synthesis. Int J Drug Policy.

[41] Health Canada. A Framework for the Legalization and Regulation of Cannabis in Canada: The Final Report of the Task Force on Cannabis Legalization and Regulation [Internet]. Ottawa (CA): Health Canada. 2016. https://www.canada.ca/en/health-canada/services/drugs-medication/cannabis/laws-regulations/task-force-cannabis-legalization-regulation/framework-legalization-regulation-cannabis-in-canada.html.

[42] Arksey H, O’Malley L (2005). Scoping studies: towards a methodological framework. Int J Soc Res Methodol.

[43] Levac D, Colquhoun H, O’Brien KK (2010). Scoping studies: advancing the methodology. Implement Sci.

[44] Kourgiantakis T, Edwards T, Lee E, Logan J, Vicknarajah R, Craig SL, Simon-Tucker M, Williams CC. Protocol: Cannabis use among youth in Canada: a scoping review protocol. BMJ Open. 2022;12(6). 10.1136/bmjopen-2022-061997 PMC9214380 35725253

[45] Tricco AC, Lillie E, Zarin W, O’Brien KK, Colquhoun H, Levac D, Moher D, Peters MD, Horsley T, Weeks L, Hempel S (2018). PRISMA extension for scoping reviews (PRISMA-ScR): checklist and explanation. Ann Intern Med.

[46] Campbell S. Hedge to retrieve studies related to Canada and Canadian provinces from most databases [Internet]. Edmonton (CA): John W. Scott Health Sciences Library, University of Alberta; 2020 Mar 9. http://guides.library.ualberta.ca/health-sciences-search-filters/geographic-filters.

[47] Campbell S, Kung JYC. Filter to retrieve studies related to cannabis in the Ovid Medline database [Internet]. Edmonton: John W. Scott Health Sciences Library, University of Alberta; 2018 Aug 21. https://docs.google.com/document/d/1PbH8GtKrGBLicFJSHRubpMpwmFsyo6oBoqx15sCm4RE/edit.

[48] Canadian Health Libraries Association (CHLA). Cannabis [Internet]. Canadian Health Libraries Association. 2018 Apr 20. https://extranet.santecom.qc.ca/wiki/!biblio3s/doku.php?id=concepts:cannabis.

[49] Tessier V, Lacourse M. Adolescents et jeunes adultes [Internet]. [place unknown]: Canadian Health Libraries Association; 2018 Dec 3. https://extranet.santecom.qc.ca/wiki/!biblio3s/doku.php?id=concepts:adolescents-et-jeunes-adultes.

[50] McGowan J, Sampson M, Salzwedel DM, Cogo E, Foerster V, Lefebvre C (2016). PRESS peer review of electronic search strategies: 2015 guideline statement. J Clin Epidemiol.

[51] Mental Health Commission of Canada (MHCC). Taking the next step forward. Building a responsive mental health and addiction system for emerging adults. 2015. https://mentalhealthcommission.ca/resource/taking-the-next-step-forward-building-a-responsive-mental-health-and-addictions-system-for-emerging-adults/.

[52] Ritchie J, Spencer L. Qualitative data analysis for applied policy research. In: Analyzing qualitative data 2002 sep 9 (pp. 173–94). Routledge.

[53] Baker MM, Procter TD, Belzak L, Ogunnaike-Cooke S (2022). Vaping-associated lung illness (VALI) in Canada: a descriptive analysis of VALI cases reported from September 2019 to December 2020. Health Promot Chronic Dis Prev Can.

[54] Buliga E, MacInnis CC (2022). Exploring associations between intergroup contact, ideology, and support for new restrictive cannabis policies and penalties in Canada. Can J Behav Sci/Can sci Comport.

[55] Callaghan RC, Vander Heiden J, Sanches M, Asbridge M, Hathaway A, Kish SJ (2021). Impacts of Canada’s cannabis legalization on police-reported crime among youth: early evidence. Addiction.

[56] Fischer B, Lee A, Robinson T, Hall W (2021). An overview of select cannabis use and supply indicators pre-and post-legalization in Canada. Subst Abuse Treat Prev Policy.

[57] Harris-Lane LM, Drakes DH, Donnan JR, Rowe EC, Bishop LD, Harris N (2023). Emerging adult perceptions of cannabis consumption post-legalization: considering age and sex differences. J Adolesc Health.

[58] Imtiaz S, Wells S, Rehm J, Wickens CM, Hamilton H, Nigatu YT, Jankowicz D, Elton-Marshall T (2022). Daily cannabis use during the novel coronavirus disease (COVID-19) pandemic in Canada: a repeated cross-sectional study from May 2020 to December 2020. Subst Abuse Treat Prev Policy.

[59] Imtiaz S, Wells S, Rehm J, Hamilton HA, Nigatu YT, Wickens CM, Jankowicz D, Elton-Marshall T (2021). Cannabis use during the COVID-19 pandemic in Canada: a repeated cross-sectional study. J Addict Med.

[60] Nguyen HV, Mital S, Bornstein S (2023). Short-term effects of recreational cannabis legalization on youth cannabis initiation. J Adolesc Health.

[61] Yousufzai SJ, Cole AG, Nonoyama M, Barakat C (2022). Changes in cannabis consumption among emerging adults in relation to policy and public health developments. Subst Use Misuse.

[62] Zutrauen S, Do MT, Ghandour L, Moore-Hepburn C, Beno S, Richmond SA, Chadi N (2022). Acute injury or illness related to the inhalation of vaping aerosols among children and adolescents across Canada: a cross-sectional survey of Canadian paediatricians. Paediatr Child Health.

[63] Magier MJ, Leatherdale ST, Wade TJ, Patte KA (2022). The relations between Youth Cannabis Use, School Cannabis Use-related disciplinary approaches and student perceptions of School Support. Subst Use Misuse.

[64] Myran DT, Tanuseputro P, Auger N, Konikoff L, Talarico R, Finkelstein Y (2023). Pediatric hospitalizations for unintentional cannabis poisonings and all-cause poisonings associated with edible cannabis product legalization and sales in Canada. JAMA Health Forum.

[65] Romano I, Butler A, Williams G, Aleyan S, Patte KA, Leatherdale ST (2022). Risky cannabis use is associated with varying modes of cannabis consumption: gender differences among Canadian high school students. Drug Alcohol Depend.

[66] Romano I, Patte KA, de Groh M, Jiang Y, Leatherdale ST. Perceptions of and adherence to early COVID-19-related restrictions and associations with substance use among youth in Canada. Health Promot Chronic Dis Prev Can. 2022:479–89. 10.24095/hpcdp.42.11/12.03 PMC9903852 36165768

[67] Williams GC, Patte KA, Ferro MA, Leatherdale ST (2021). Associations between longitudinal patterns of substance use and anxiety and depression symptoms among a sample of Canadian secondary school students. J Environ Res Public Health.

[68] Yang Y, Butt ZA, Leatherdale ST, Morita PP, Wong A, Rosella L, Chen HH. Exploring the dynamic transitions of polysubstance use patterns among Canadian youth using Latent Markov models on COMPASS data. Lancet Reg Health - Am. 2022;16. 10.1016/j.lana.2022.100389 PMC9904069 36777157

[69] Zuckermann AM, Battista KV, Bélanger RE, Haddad S, Butler A, Costello MJ, Leatherdale ST (2021). Trends in youth cannabis use across cannabis legalization: data from the COMPASS prospective cohort study. Prev Med Rep.

[70] Zuckermann AM, Gohari MR, Romano I, Leatherdale ST (2021). Changes in cannabis use modes among Canadian youth across recreational cannabis legalization: data from the COMPASS prospective cohort study. Addict Behav.

[71] Leatherdale ST, Bélanger RE, Gansaonré RJ, Patte KA, deGroh M, Jiang Y, Haddad S (2021). Examining the impact of the early stages of the COVID-19 pandemic period on youth cannabis use: adjusted annual changes between the pre-COVID and initial COVID-lockdown waves of the COMPASS study. BMC Public Health.

[72] Callaghan RC, Sanches M, Vander Heiden J, Asbridge M, Stockwell T, Macdonald S, Peterman BH, Kish SJ (2021). Canada’s cannabis legalization and drivers’ traffic-injury presentations to emergency departments in Ontario and Alberta, 2015–2019. Drug Alcohol Depend.

[73] MacDougall C, Maston M (2021). Student perceptions of cannabis use. J Am Coll Health.

[74] Andrews CN, Rehak R, Woo M, Walker I, Ma C, Forbes N, Rittenbach K, Hathaway J, Wilsack L, Liu A, Nasser Y (2022). Cannabinoid hyperemesis syndrome in North America: evaluation of health burden and treatment prevalence. Aliment Pharmacol Ther.

[75] Hammond D, Goodman S, Wadsworth E, Freeman TP, Kilmer B, Schauer G, Pacula RL, Hall W. 2022. Trends in the use of cannabis products in Canada and the USA, 2018–2020: Findings from the International Cannabis Policy Study. Int J Drug Policy. 2022;105:103716. 10.1016/j.drugpo.2022.103716 35613480

[76] Hammond D, Wadsworth E, Reid JL, Burkhalter R (2021). Prevalence and modes of cannabis use among youth in Canada, England, and the US, 2017 to 2019. Drug Alcohol Depend.

[77] Coret A, Rowan-Legg A (2022). Unintentional cannabis exposures in children pre-and post-legalization: a retrospective review from a Canadian paediatric hospital. Paediatr Child Health.

[78] Dumas TM, Ellis W, Litt DM (2020). What does adolescent substance use look like during the COVID-19 pandemic? Examining changes in frequency, social contexts, and pandemic-related predictors. J Adolesc Health.

[79] Hawke LD, Henderson J (2021). Legalization of cannabis use in Canada: impacts on the cannabis use profiles of youth seeking services for substance use. J Subst Abuse Treat.

[80] Jani P, Song N, Artna E, Lyeo J, Assam A, Maelzer F, Murphy A, Grant A, Archie S. Online knowledge translation program involving video games and university student–led tutorials about cannabis and psychosis for black youth: mixed method feasibility study. JMIR Form Res 2022 Mar 17:33693–721. 10.2196/33693 PMC9253977 35315782

[81] Kim S, Rajack N, Mondoux SE, Tardelli VS, Kolla NJ, Le Foll B (2023). The COVID-19 impact and characterization on substance use‐related emergency department visits for adolescents and young adults in Canada: practical implications. J Eval Clin Pract.

[82] Moreno G, Van Mierlo T (2021). A digital health tool to understand and prevent cannabis-impaired driving among youth: a cross-sectional study of responses to a brief intervention for cannabis use. JMIR Form Res.

[83] Myran DT, Pugliese M, Tanuseputro P, Cantor N, Rhodes E, Taljaard M. The association between recreational cannabis legalization, commercialization and cannabis-attributable emergency department visits in Ontario, Canada: an interrupted time–series analysis. Volume 117. Addiction; 2022. pp. 1952–60. 10.1111/add.15834 35170149

[84] Myran DT, Roberts R, Pugliese M, Taljaard M, Tanuseputro P, Pacula RL. Changes in emergency department visits for cannabis hyperemesis syndrome following recreational cannabis legalization and subsequent commercialization in Ontario, Canada. JAMA Netw Open, 5 (9) (2022), Article e2231937. 10.1001/jamanetworkopen.2022.31937 PMC9482056 36112372

[85] Sampasa-Kanyinga H, Bakwa-Kanyinga F, Hamilton HA, Chaput JP (2022). Cyberbullying involvement, parental support, and cannabis use among adolescents. Child Abuse Negl.

[86] Zhang EW, Davis A, Finkelstein Y, Rosenfield D. The effects of COVID-19 on intoxications in the pediatric emergency room. Paediatr Child Health. 2022:6. 10.1093/pch/pxab100 PMC9126273 35620562

[87] Auger N, Luu TM, Ayoub A, Bilodeau-Bertrand M, Lo E, Low N (2021). Cannabis-related hospitalizations among youth in Canada before and after cannabis legalization. J Addict Med.

[88] Gueye ND, Prada K, de Moissac D (2021). Pre- and post-recreational cannabis legislation: snapshot of postsecondary student cannabis use in Manitoba, Canada. Can J Addict.

[89] Salmon S, Taillieu TL, Stewart-Tufescu A, MacMillan HL, Tonmyr L, Gonzalez A, Afifi TO (2022). Stressors and symptoms associated with a history of adverse childhood experiences among older adolescents and young adults during the COVID-19 pandemic in Manitoba, Canada. Can Health Promot Chronic Dis Prev Can.

[90] Robinson JM, Copeland C, Pilin MA, Meyer A, Krank MD (2020). The Impact of Cannabis Legalization in Canada on adolescents’ perceptions. J Drug Issues.

[91] Turuba R, Amarasekera A, Howard AM, Brockmann V, Tallon C, Irving S, Mathias S, Henderson J, Marchand K, Barbic S (2022). A qualitative study exploring how young people perceive and experience substance use services in British Columbia, Canada. Subst Abuse Treat Prev Policy.

[92] Hammami N, Katapally TR (2022). Do associations between suicide ideation and its correlates (substance use, anxiety, and depression) differ according to victimization type among youth? A smart platform study. Prev Med Rep.

[93] Yeung ME, Weaver CG, Hartmann R, Haines-Saah R, Lang E (2021). Emergency department pediatric visits in Alberta for cannabis after legalization. Pediatrics.

[94] Bishop LD, Drakes DH, Donnan JR, Rowe EC, Najafizada M. Exploring youths’ Cannabis Health literacy Post legalization: a qualitative study. J Adolesc Res 2022:07435584221118380. 10.1177/07435584221118380 PMC11637917 39678432

[95] Butler A, Doggett A, Vermeer J, Magier M, Patte KA, Maginn D, Markham C, Leatherdale ST (2022). Shifting school health priorities pre–post cannabis legalization in Canada: Ontario secondary school rankings of student substance use as a health-related issue. Health Educ Res.

[96] Paschall MJ, Grube JW (2020). Recreational marijuana availability in Oregon and use among adolescents. Am J Prev Med.

[97] Laqueur H, Rivera-Aguirre A, Shev A, Castillo-Carniglia A, Rudolph KE, Ramirez J, Martins SS, Cerdá M (2020). The impact of cannabis legalization in Uruguay on adolescent cannabis use. Int J Drug Policy.

[98] Myran DT, Friesen EL, Dickson S, Konikoff L, Arora G, Tanuseputro P (2023). Access to legal cannabis market in Canada over the four years following non-medical cannabis legalisation. Drug Alcohol Rev.

[99] Gouvernement du Québec. Loi encadrant le cannabis [Internet]. Québec (CA): Gouvernement du Québec. 2023. https://encadrementcannabis.gouv.qc.ca/loi/loi-encadrant-le-cannabis/.

[100] Ali F, Russell C, Nafeh F, Chaufan C, Imtiaz S, Rehm J (2022). Youth substance use service provider’s perspectives on use and service access in Ontario: time to reframe the discourse. Subst Abuse Treat Prev Policy.

[101] Parker H, Williams L, Aldridge J (2002). The normalization of ‘Sensible’ recreational drug use: further evidence from the North West England Longitudinal Study. Sociology.

[102] Asbridge M, Valleriani J, Kwok J, Erickson P (2016). Normalization and denormalization in different legal contexts: comparing cannabis and tobacco. Drugs: Educ Prev Policy.

[103] Canadian Centre on Substance Use and Addiction (CCSA). A public health perspective on cannabis legalization and regulation in Canada [Internet]. Ottawa (CA): Canadian Centre on Substance Use and Addiction. 2023. https://www.ccsa.ca/sites/default/files/2023-01/CCSA_Cannabis_Act_Legislative_review_update_l_en.pdf.

[104] Kourgiantakis T, Lee E, Kosar AK, Tait C, Lau CK, McNeil S, Craig S, Ashcroft R, Williams CC, Goldstein AL, Chandrasekera U (2023). Youth cannabis use in Canada post-legalization: service providers’ perceptions, practices, and recommendations. Subst Abuse Treat Prev Policy.

[105] Health Canada. Legislative review of the Cannabis Act. Final report of the expert panel. 2024. https://www.canada.ca/en/health-canada/services/publications/drugs-medication/legislative-review-cannabis-act-final-report-expert-panel.html.

[106] Callaghan RC, Sanches M, Hathaway A, Asbridge M, MacDonald M, Kish SJ. Canada’s cannabis legalization and police-reported cannabis-related criminal incidents among youth, 2015–2021. Drug and alcohol dependence. 2023 Apr 23:109892. 10.1016/j.drugalcdep.2023.109892 37183068

[107] Owusu-Bempah A, Rehmatullah T. Waiting to Inhale: Cannabis Legalization and the fight for racial justice. MIT Press; 2023 Apr. p. 11.

