# Supplementary material for: Understanding youth and young adult cannabis use in Canada post-legalization: a scoping review on a public health issue
Source: Subst Abuse Treat Prev Policy. 2024 Jun 17;19:30. doi: 10.1186/s13011-024-00615-9 (PMC11184772; doi:10.1186/s13011-024-00615-9)
Supplement: Supplementary file 2 — Supplementary Material 2 [file 13011_2024_615_MOESM2_ESM.docx]

**Appendix B**

**Full Electronic Search Strategy for Five Databases**

Results from the following searches were downloaded on December 8, 2021.

## **Medline:** Epub Ahead of Print, In-Process & Other Non-Indexed Citations, Ovid MEDLINE® Daily and Ovid MEDLINE

| 1. | (canad* or "british columbia*" or "Colombie britannique" or alberta* or saskatchewan* or manitoba* or ontari* or quebec* or "nouveau brunswick" or "nova scotia*" or "nouvelle ecosse" or "prince edward island*" or newfoundland* or labrador* or nunavu* or nwt or "northwest territori*" or yukon* or nunavik or inuvialuit).tw,kf,jw,nw. |
| --- | --- |
| 2. | exp Canada/ |
| 3. | 1 or 2 [Canada] |
| 4. | "delta(9)-tetrahydrocannabinolic acid".nm. or (cannabi* or dronabinol or ha?chi?ch$1 or hashis?h$1 or hash or mari#uana* or tetrahydrocannabi* or tetra hydro cannabi*or THC or bhang or bhangs or cesamet or dexanabinol or dronabinol or ganja or ganjas or hemp or marinol or nabilone or nabiximol* or sativex or indica or sativa or CBD).tw,kf. or (tetrahydro adj cannabi*).tw,kf. or ((blunt or blunts or pot) adj2 smok*).tw,kf. |
| 5. | exp cannabinoids/ or cannabis/ or "marijuana abuse"/ or exp "marijuana use"/ or "medical marijuana"/ or "cannabinoid receptor agonists"/ or "marijuana abuse"/ |
| 6. | 4 or 5 [Cannabis] |
| 7. | (teen* or youth* or adolescen* or child* or puberty or pubescen* or juvenile* or (young adj2 (adult* or person* or individual* or people* or population* or man or men or wom#n)) or youngster* or highschool* or college* or universit* or ((secondary or high*) adj2 (school* or education)) or "emerging adult*").tw,kf. |
| 8. | adolescent/ or young adult/ or child/ or Adolescent Health Services/ |
| 9. | 7 or 8 [Youth] |
| 10. | 3 and 6 and 9 |
| 11. | limit 10 to yr="2000 -Current" |

## **Embase Classic+Embase**

| 1. | (canad* or "british columbia*" or "Colombie britannique" or alberta* or saskatchewan* or manitoba* or ontari* or quebec* or "nouveau brunswick" or "nova scotia*" or "nouvelle ecosse" or "prince edward island*" or newfoundland* or labrador* or nunavu* or nwt or "northwest territori*" or yukon* or nunavik or inuvialuit).tw,kf,jx. |
| --- | --- |
| 2. | exp Canada/ |
| 3. | 1 or 2 [Canada] |
| 4. | "delta9 11 tetrahydrocannabinol".du. or (cannabi* or dronabinol or ha?chi?ch$1 or hashis?h$1 or hash or mari#uana* or tetrahydrocannabi* or tetra hydro cannabi*or THC or bhang or bhangs or cesamet or dexanabinol or dronabinol or ganja or ganjas or hemp or marinol or nabilone or nabiximol* or sativex or indica or sativa or CBD).tw,kf. or (tetrahydro adj cannabi*).tw,kf. or ((blunt or blunts or pot) adj2 smok*).tw,kf. |
| 5. | "cannabis smoking"/ or exp cannabinoid/ or "cannabis use"/ |
| 6. | 4 or 5 [Cannabis] |
| 7. | (teen* or youth* or adolescen* or child* or puberty or pubescen* or juvenile* or (young adj2 (adult* or person* or individual* or people* or population* or man or men or wom#n)) or youngster* or highschool* or college* or universit* or ((secondary or high*) adj2 (school* or education)) or "emerging adult*").tw,kf. |
| 8. | exp adolescent/ or exp adolescence/ or young adult/ |
| 9. | 7 or 8 [Youth] |
| 10. | 3 and 6 and 9 |
| 11. | limit 10 to yr="2000 -Current" |

## **APA PsycINFO**

| 1. | (canad* or "british columbia*" or "Colombie britannique" or alberta* or saskatchewan* or manitoba* or ontari* or quebec* or "nouveau brunswick" or "nova scotia*" or "nouvelle ecosse" or "prince edward island*" or newfoundland* or labrador* or nunavu* or nwt or "northwest territori*" or yukon* or nunavik or inuvialuit).tw,jx. |
| --- | --- |
| 2. | ("delta(9)-tetrahydrocannabinolic acid" or cannabi* or dronabinol or ha?chi?ch$1 or hashis?h$1 or hash or mari#uana* or tetrahydrocannabi* or tetra hydro cannabi* or THC or bhang or bhangs or cesamet or dexanabinol or dronabinol or ganja or ganjas or hemp or marinol or nabilone or nabiximol* or sativex or indica or sativa or CBD or (tetrahydro adj cannabi*) or ((blunt or blunts or pot) adj2 smok*)).tw. |
| 3. | cannabinoids/ or cannabis/ or marijuana/ or "marijuana usage"/ or "marijuana legalization"/ or "marijuana laws"/ or tetrahydrocannabinol/ or hashish/ |
| 4. | 2 or 3 [Cannabis] |
| 5. | (teen* or youth* or adolescen* or child* or puberty or pubescen* or juvenile* or (young adj2 (adult* or person* or individual* or people* or population* or man or men or wom#n)) or youngster* or highschool* or college* or universit* or ((secondary or high*) adj2 (school* or education)) or "emerging adult*").tw. |
| 6. | "Adolescent Health"/ or "Emerging adulthood"/ or "Early Adolescence"/ or Puberty/ or "Adolescent Development"/ or "Childhood Development"/ or "child health"/ |
| 7. | 5 or 6 [Youth] |
| 8. | 1 and 4 and 7 |
| 9. | limit 8 to yr="2000 -Current" |

## **CINAHL**

| **#** | **Query** | **Limiters** |
| --- | --- | --- |
| S1 | TI ( (canad* OR "british columbia" OR "Colombie britannique" OR alberta* OR saskatchewan* OR manitoba* OR ontari* OR quebec* OR "nouveau brunswick" OR "nova scotia*" OR "nouvelle ecosse" OR "prince edward island*" OR newfoundland* OR labrador* OR nunavu* OR nwt OR "northwest territori*" OR yukon* OR nunavik OR inuvialuit) ) OR AB ( (canad* OR "british columbia" OR "Colombie britannique" OR alberta* OR saskatchewan* OR manitoba* OR ontari* OR quebec* OR "nouveau brunswick" OR "nova scotia*" OR "nouvelle ecosse" OR "prince edward island*" OR newfoundland* OR labrador* OR nunavu* OR nwt OR "northwest territori*" OR yukon* OR nunavik OR inuvialuit) ) or SO ( (canad* OR "british columbia" OR "Colombie britannique" OR alberta* OR saskatchewan* OR manitoba* OR ontari* OR quebec* OR "nouveau brunswick" OR "nova scotia*" OR "nouvelle ecosse" OR "prince edward island*" OR newfoundland* OR labrador* OR nunavu* OR nwt OR "northwest territori*" OR yukon* OR nunavik OR inuvialuit) ) OR (MH "Canada+") |  |
| S2 | TI ( "delta(9)-tetrahydrocannabinolic acid" or cannabi* or dronabinol or ha#chi#ch* or hashis#h* or hash or mari?uana* or tetrahydrocannabi* or "tetra hydro cannabi*" or THC or bhang or bhangs or cesamet or dexanabinol or dronabinol or ganja or ganjas or hemp or marinol or nabilone or nabiximol* or sativex or indica or sativa or CBD or (tetrahydro N1 cannabi*) or ((blunt or blunts or pot) N2 smok*) ) OR AB ( "delta(9)-tetrahydrocannabinolic acid" or cannabi* or dronabinol or ha#chi#ch* or hashis#h* or hash or mari?uana* or tetrahydrocannabi* or "tetra hydro cannabi*" or THC or bhang or bhangs or cesamet or dexanabinol or dronabinol or ganja or ganjas or hemp or marinol or nabilone or nabiximol* or sativex or indica or sativa or CBD or (tetrahydro N1 cannabi*) or ((blunt or blunts or pot) N2 smok*) ) OR ( MH ("Cannabis+" OR "Medical Marijuana") ) |  |
| S3 | TI ( (teen* or youth* or adolescen* or child* or puberty or pubescen* or juvenile* or (young N2 (adult* or person* or individual* or people* or population* or man or men or wom?n)) or youngster* or highschool* or college* or universit* or ((secondary or high*) N2 (school* or education)) or "emerging adult*") ) OR AB ( (teen* or youth* or adolescen* or child* or puberty or pubescen* or juvenile* or (young N2 (adult* or person* or individual* or people* or population* or man or men or wom?n)) or youngster* or highschool* or college* or universit* or ((secondary or high*) N2 (school* or education)) or "emerging adult*") ) OR MH ( Adolescence OR "Young adult" ) |  |
| S4 | S1 AND S2 AND S3 | Published Date: 20000101- |

## **Web of Science Core Collection**

Includes: Science Citation Index Expanded, Social Sciences Citation Index, Arts & Humanities Citation Index, Emerging Sources Citation Index, Conference Proceedings Citation Index, and Book Citation Index

| #1 | (TS=(canad* OR "british columbia*" OR "Colombie britannique" OR alberta* OR saskatchewan* OR manitoba* OR ontari* OR quebec* OR "nouveau brunswick" OR "nova scotia*" OR "nouvelle ecosse" OR "prince edward island*" OR newfoundland* OR labrador* OR nunavu* OR nwt OR "northwest territori*" OR yukon* OR nunavik OR inuvialuit)) OR SO=(canad* OR "british columbia*" OR "Colombie britannique" OR alberta* OR saskatchewan* OR manitoba* OR ontari* OR quebec* OR "nouveau brunswick" OR "nova scotia*" OR "nouvelle ecosse" OR "prince edward island*" OR newfoundland* OR labrador* OR nunavu* OR nwt OR "northwest territori*" OR yukon* OR nunavik OR inuvialuit) |
| --- | --- |
| #2 | TS=("delta(9)-tetrahydrocannabinolic acid" or cannabi* or dronabinol or ha$chi* or hashis$h* or hash or mari?uana* or tetrahydrocannabi* or "tetra hydro cannabi*" or THC or bhang or bhangs or cesamet or dexanabinol or dronabinol or ganja or ganjas or hemp or marinol or nabilone or nabiximol* or sativex or indica or sativa or CBD or (tetrahydro NEAR/1 cannabi*) or ((blunt or blunts or pot) NEAR/2 smok*)) |
| #3 | TS=(teen* or youth* or adolescen* or child* or puberty or pubescen* or juvenile* or (young NEAR/2 (adult* or person* or individual* or people* or population* or man or men or wom?n)) or youngster* or highschool* or college* or universit* or ((secondary or high*) NEAR/2 (school* or education)) or "emerging adult*") |
| #4 | (#1 and #2 and #3) AND PY=(2000-2023) |
